# Simulating the chromatin-mediated phase separation of model proteins with multiple domains

**DOI:** 10.1016/j.bpj.2022.05.039

**Published:** 2022-05-28

**Authors:** Marco Ancona, Chris A. Brackley

**Affiliations:** 1 SUPA, School of Physics and Astronomy, University of Edinburgh, Peter Guthrie Tait Road, Edinburgh, United Kingdom

## Abstract

We perform simulations of a system containing simple model proteins and a polymer representing chromatin. We study the interplay between protein-protein and protein-chromatin interactions, and the resulting condensates that arise due to liquid-liquid phase separation, or a via a “bridging-induced attraction” mechanism. For proteins that interact multivalently, we obtain a phase diagram which includes liquid-like droplets, droplets with absorbed polymer, and coated polymer regimes. Of particular interest is a regime where protein droplets only form due to interaction with the polymer; here, unlike a standard phase separating system, droplet density rather than size varies with the overall protein concentration. We also observe that protein dynamics within droplets slow down as chromatin is absorbed. If the protein-protein interactions have a strictly limited valence, fractal or gel-like condensates are instead observed. A specific example that inspired our model is heterochromatin protein 1, or HP1. Recent in vivo experiments have shown that HP1 exhibits similar droplet size buffering behavior as our simulations. Overall, our results provide biologically relevant insights into the general nature of protein-chromatin condensates in living cells.

## Significance

Liquid-liquid phase separation has been much discussed as a mechanism for protein droplet formation in the cell nucleus. Yet how this can drive gene-regulatory protein clustering only at specific chromatin sites remains unknown. Here, we study the physics of clustering of chromatin binding proteins using simple simulation models. We focus on the interplay between protein self-interactions and protein-chromatin bridging. Our results on cluster structure, dynamics, and polymer compaction, have strong implications for our understanding of the role these processes play in vivo. Particularly, we find that chromatin acts to slow down protein dynamics within droplets, and we uncover a regime where varying the overall protein concentration alters the concentration within and outside the droplet, strikingly different to standard phase separation.

## Introduction

The cell nucleus is a highly structured organelle, which contains much of an organism’s genetic material (1). This material exists as chromatin, a composite of DNA and histone proteins that makes up the chromosomes. While the nucleus itself is surrounded by a membrane, most of the structures within it (known as “nuclear bodies” (2)) are membraneless assemblies of proteins, DNA and/or RNA. Some of these, including nucleoli, Cajal bodies, and splicing speckles, are found in the interchromatin regions. Others colocalize with chromatin, and examples include: clusters of transcription factors, RNA polymerase II, and other proteins associated with transcription (3, 4); polycomb bodies, involved in cell-type-specific gene repression (5, 6); and foci of heterochromatin, a tightly packaged form of chromatin which tends to be transcriptionally repressed (7, 8).

There has been much recent interest in how protein foci form in the nucleus, and whether a liquid-liquid phase separation (LLPS) mechanism plays a role. A common notion is that flexible, low complexity, and intrinsically disordered protein (IDP) domains facilitate LLPS (9). IDPs often contain exposed charges or hydrophobic residues, leading to weak multivalent attractive interactions; having multiple interaction points and a “coil” configuration is thought to lead to interactions that are effectively longer ranged than those between globular proteins (10). Many IDPs, and several proteins that possess both disordered and globular domains, have indeed been found to readily phase separate in vitro. There has been much recent experimental and simulation work on mixtures of multiple protein species (11, 12, 13) and mixtures of proteins and RNA (14, 15). Often in these mixtures there are “scaffold” species, which can phase separate on their own, and “client” species, which do not phase separate when in isolation, but absorb into scaffold condensates increasing or disrupting their stability.

For the case of chromatin binding proteins, which we consider here, another mechanism that can lead to protein phase separation is the “bridging-induced attraction” (BIA). This was first uncovered in simulations studying how protein-chromatin interactions can drive chromosome organization (16, 17), and was more recently demonstrated in vitro (18). It arises when proteins or protein complexes with multiple DNA/chromatin binding domains form molecular bridges between different chromatin regions. The first protein to form a bridge produces a local increase in chromatin density, which leads to further protein binding and bridging at that location; this positive feedback ultimately gives rise to protein clustering. For the case of proteins that bind nonspecifically to any chromatin site, the clusters will grow and coarsen until a single protein-rich phase remains (19); when there is an excess of proteins this also leads to chromatin compaction (20). Importantly, the BIA can give rise to phase separated foci in the absence of protein-protein interactions; we call this bridging-induced phase separation (BIPS). In (21) it was shown that, for model proteins with a finite number of chromatin binding domains, the shape of the protein can determine its ability to form bridges: for proteins that readily form bridges (“good bridgers”), the BIA is in effect and there is strong clustering and compaction. For poor bridgers, the BIA is not (or is only weakly) in effect, and protein clustering is not observed.

The idea that LLPS is involved in genome regulation gained popularity after it was shown that heterochromatin protein 1 (HP1), one of the chief constituents of heterochromatin, was found to undergo phase separation in vitro (22, 23). HP1 is highly conserved in eukaryotes, and is known to colocalize with heterochromatin foci (24). Its exact function in heterochromatin formation and gene silencing, however, remains elusive; possibilities are that it directly drives chromatin compaction, that it sterically occludes binding of activating proteins, or that it recruits further gene silencing machinery (25, 26). In mammals there are three paralogs: HP1α and HP1β are thought to have distinct roles in heterochromatin function, while HP1γ also has a function in active chromatin (27). All have a similar structure, with two globular domains and three flexible/disordered regions. In the nucleus, HP1 is mainly found in dimers (27, 26), which have two chromatin binding domains, and so these can in principle form bridges.

In this paper we study the interplay between LLPS and BIPS, considering how they could drive protein-chromatin foci localization and compaction in vivo. Inspired by work on patchy particles (11, 12, 13, 14, 28, 29, 30, 31, 32), we have developed a simple coarse-grained model protein that resembles HP1, and we simulate these in solution with a chromatin fiber. More specifically, we consider two separate models that mimic two microscopic possibilities: 1) that the low-complexity domains give rise to weak and longer-ranged multivalent protein-protein attractions; and 2) that the interactions between flexible domains are short ranged and have a limited valence such that exactly two domains can interact at a time. The first case involves a scenario where the flexible domains adopt an extended coil configuration, meaning that multiple coils can overlap and there will be multiple weakly interacting contact points. The second case could arise, for example, when a disordered protein domain forms a globular secondary structure when interacting with the correct binding partner (33). We explore the parameter space of the two systems to understand under what conditions aggregates containing both proteins and chromatin form, and measure the structural and dynamical properties. Importantly, our scheme is simple enough to allow us to perform simulations at many different points in parameter space, but retains details of the domain structure of the protein (explicitly incorporating protein-protein and protein-DNA interaction domains). Although the model is inspired by HP1, due to its simplicity we expect our results to be applicable more widely.

## Methods

In this work we use coarse-grained Langevin dynamics simulations to study the behavior of a system of simple HP1-inspired model proteins interacting with a model chromatin fiber.

### Chromatin polymer model

For chromatin we use a common coarse-grained polymer model where the fiber is represented as a chain of beads of diameter σ=10 nm, representing roughly 1 kbp of DNA or four to five nucleosomes. The beads interact sterically with each other via a Weeks-Chandler-Anderson potential, and they are connected via finitely extensible nonlinear elastic springs; a Kratky-Porod potential provides bending rigidity. The large-scale physical properties of the fiber are represented (i.e., its flexibility), but not the internal nucleosome structure.

### HP1 model

**Figure 1:**
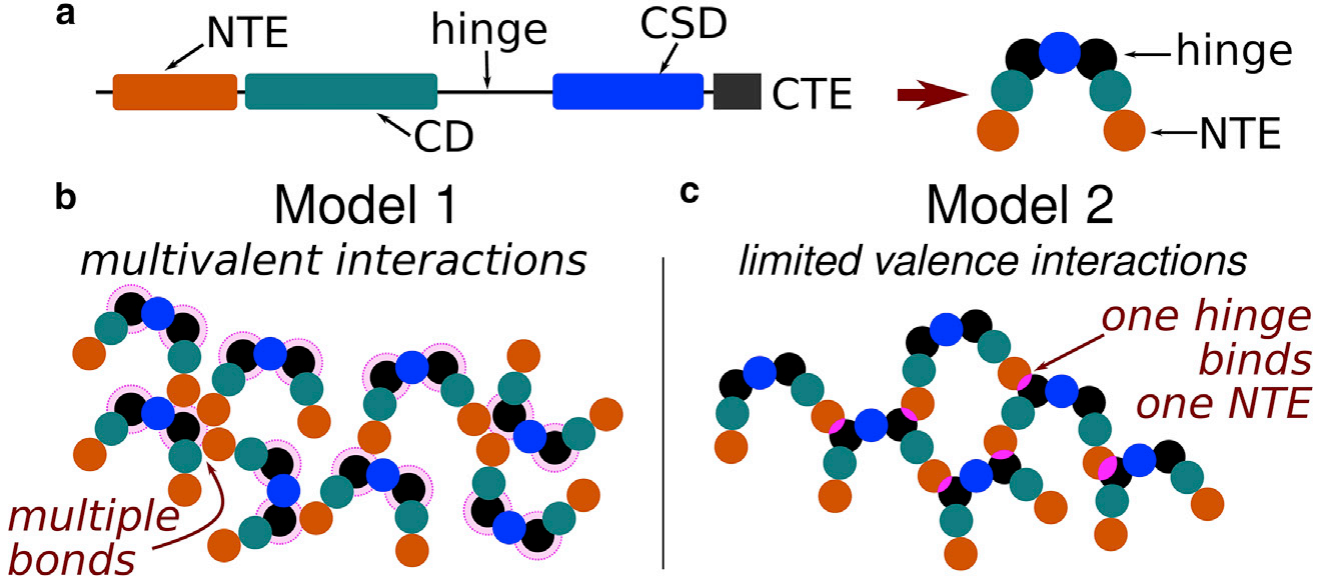
A simple coarse-grained protein model inspired by HP1. (*a*) Left: schematic showing the domain structure of the HP1 protein as detailed in the text. Right: schematic representation of the model HP1 dimer. (*b* and *c*) Two alternative models for interactions between HP1 dimers. To see this figure in color, go online.

Fig. 1*a* shows a schematic representation of the domains of HP1 (as detailed in, e.g., (22, 26)). These are known as the C-terminal end, the chromoshadow domain (CSD), a “hinge” region, the chromodomain (CD), and the N-terminal end (NTE). The CSD and CD are globular domains, while the others are flexible. Two HP1s form a dimer across the CSD, and the CD interacts with chromatin by binding trimethylated lysines in the H3 histone (H3K9me3, a histone posttranslational modification, which is a hallmark of heterochromatin). In (22) it was shown that, in human HP1, interaction between the hinge and the (phosphorylated) NTE allows further oligomerization and, eventually, phase separation. Fig. 1 *a* also shows a schematic of how HP1 dimers (which we hereon refer to as HP1s) are represented in our simulations. They are modeled as rigid bodies made up from seven spheres, each representing a different domain (the NTEs, CDs, and hinges within each dimer are represented by one sphere each, and a seventh sphere represents the two CSDs bound together; see also Fig. S1 in the supporting material). Our coarse-grained approach does not attempt to model the full details and exact dimensions of the dimer; nevertheless, we aim to capture the main features of the physics at the mesoscale. For simplicity, all HP1 component spheres have a diameter 0.5*σ*, which gives a rough size of 1–1.5*σ* ≈ 10–15 nm for a dimer (compared with 13–22 nm for a real HP1 dimer (22)). Spheres representing CDs interact with polymer beads attractively, while all others interact with the polymer sterically only. Steric interactions are provided by a Weeks-Chandler-Anderson potential, while attractive interactions are provided by a potential with a functional form similar to the Morse potential. Full details of the HP1 geometry and all interaction potentials are given in supporting material.

As noted above, we study two versions of the model that differ in their protein-protein interactions. First, we consider *multivalent interactions* (Fig. 1 *b*), using a longer range interaction potential between the spheres representing the hinge and NTE, such that several NTEs can simultaneously interact with a hinge and vice versa (determined by the geometry and steric hindrance). Second, we consider *limited valence interactions* (Fig. 1 *c*), using a shorter-ranged potential such that at most one hinge and one NTE can interact at a time. Since an HP1 dimer has two hinges and two NTEs, in the limited valence model a given dimer can bind to at most four others at once. Importantly, the interaction between the proteins and chromatin is identical for the two models. The strength of attractive interactions between HP1s, and between HP1s and chromatin, are given by the energies $\varepsilon_{\text{HH}}$ and $\varepsilon_{\text{HC}}$, respectively. Since the interaction potentials differ, these two values should not be compared directly (nor should $\varepsilon_{\text{HH}}$ values for the two different models). We note also that, due to the complex geometry, the quoted energy values do not necessarily represent the true minima of the interaction potential—see supporting material, section 4.

### Simulation scheme

The dynamics of the polymer beads and HP1s (rigid body translation and rotation) are governed by a Langevin equation; we perform extensive simulations using the LAMMPS molecular dynamics software (34). The dynamics are integrated using a velocity-Verlet algorithm with a time step of 0.001τ, where *τ* is a simulation time unit. All results are obtained by averaging of at least four simulations of at least $5\times 10^{3}\tau$. Further details are given in supporting material.

Below we present simulations of a system containing N=1000 model HP1s and an L=1000 bead polymer. For simplicity, we consider a homogeneous polymer where all beads can bind HP1, i.e., it represents a section of H3K9me3-modified chromatin. For the multivalent HP1 model we ran long simulations to obtain equilibrium configurations, as detailed in supporting material; in several cases we ran additional test simulations to check that the configurations are indeed representative of equilibrium, supporting material, section 5. For the limited valence HP1s, the system displayed long-lived nonequilibrium metastable states (see below). We confine all components of the system in a cubic box of size $l_{x}=35\sigma$ (approximately equal to the radius of gyration of the polymer as predicted by the worm-like chain model) by including a “wall potential.” While the confinement reduces the entropy of the system by forbidding some extended polymer configurations, it also prevents the polymer from interacting with its periodic image (test simulations showed that, in the presence of periodic boundaries, HP1 could bridge distant chromatin regions across the boundaries, and the polymer could become trapped in extended unphysical configurations).

## Results and discussion

### Model 1: Multivalent protein-protein interactions

**Figure 2:**
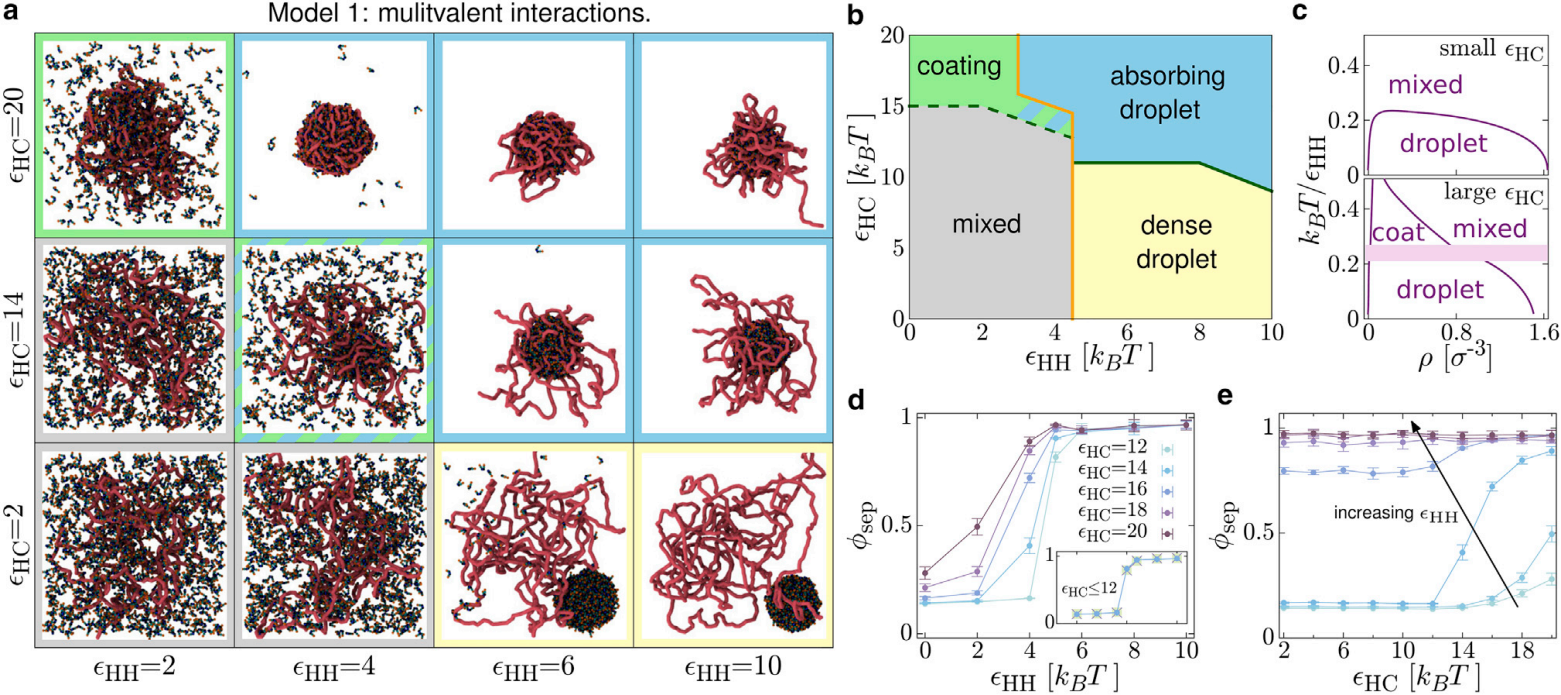
Protein-chromatin and multivalent protein-protein interactions lead to phase separation. (*a*) Snapshots of equilibrium configurations for N=1000 multivalent HP1 dimers interacting with an L=1000 bead polymer representing a 1 Mbp chromosome fragment for different values of the HP1-HP1 and HP1-chromatin interaction energies. Energies are given in units of *k*_*B*_*T*. (*b*) Phase diagram showing the different behaviors of the multivalent HP1s with different parameter values. Border colors in (*a*) indicate the relevant region in (*b*). (*c*) Phase diagram on the *ρ*-$\varepsilon_{\text{HH}}$ plane for small $\varepsilon_{\text{HC}}$ (*top*) and large $\varepsilon_{\text{HC}}$ (*bottom*). *ρ* is the total number density of HP1s. In the bottom plot the shaded bar covers a region where there is a chromatin-associated droplet, but the protein density inside and outside the droplet depends on the overall protein density (see text). These are sketch plots based on measurements of the HP1 density inside and outside of droplet (see supporting material, section 6 and Fig. S5). (*d*) Phase separation depth $\varphi_{\text{sep}}$ is plotted as a function of $\varepsilon_{\text{HH}}$ for different values of $\varepsilon_{\text{HC}}$ as indicated (units are $k_{B}T$). Each point is obtained from an average over four simulations of duration $5\times 10^{3}\tau$ (*τ* is the simulation time unit). Error bars show standard error in the mean; lines are a guide to the eye. The inset shows a similar plot for $\varepsilon_{\text{HC}}=$ 2, 6, 10, and 12 $k_{B}T$ where points overlap. (*e*) $\varphi_{\text{sep}}$ is plotted as a function of $\varepsilon_{\text{HC}}$ for $\varepsilon_{\text{HH}}$= 0, 2, 4, 5, 6, 8, and10 $k_{B}T$ (darker colors for larger values, as indicated by the arrow). To see this figure in color, go online.

With this version of the model, when the HP1-chromatin interaction energy, $\varepsilon_{\text{HC}}$, is small the proteins behave like a standard phase separating system (model B (35, 36)). When $\varepsilon_{\text{HC}}$ is larger there is more interesting behavior. We summarize the emerging regimes in the simulation snapshots and phase diagram in Fig. 2, *a*–*c*.

When $\varepsilon_{\text{HC}}<12k_{B}T$, a phase transition between a uniform mixed phase and a separated phase takes place as $\varepsilon_{\text{HH}}$ increases. Above a critical $\varepsilon_{\text{HH}}$ value a roughly spherical cluster, or “droplet,” of HP1 forms. We call this the *dense droplet* regime. By measuring the density *ρ* of HP1s inside and outside of the droplet, we can also map out the phase diagram on the *ρ*-$\varepsilon_{\text{HH}}$ plane (Fig. 2 *c* *top*, see supporting material, section 6 for details).

For small HP1-HP1 interaction energies, $\varepsilon_{\text{HH}}<5k_{B}T$, there is no droplet. Increasing the HP1-chromatin attraction leads to HP1s becoming bound to the polymer, and there is a smooth increase of the fraction bound as $\varepsilon_{\text{HC}}$ increases. For large $\varepsilon_{\text{HC}}$ there are sufficient HP1s bound such that the region occupied by the chromatin has a higher than average protein density, and the surroundings have a lower than average protein density (green region in Fig. 2, *a* and *b*). In this sense there is a phase separation; however, this regime is profoundly different from the dense droplet phase: a significant fraction of the proteins remain unbound, while the rest tend to “coat” the polymer. Hence, we refer to it as the *coating* regime.

When both $\varepsilon_{\text{HH}}$ and $\varepsilon_{\text{HC}}$ are large (*blue region* in Fig. 2, *a* and *b*) a protein droplet forms, but now the polymer is also absorbed into it. Or in other words, the droplet compacts the polymer. We call this the *absorbing droplet* regime. Interestingly, the polymer is absorbed to a different degree depending on the precise values of the interaction energies (compare snapshots at $\varepsilon_{\text{HC}}=20k_{B}T$ and different $\varepsilon_{\text{HH}}$ in Fig. 2 *a*, where different amounts of chromatin extend out from the droplet). As before, measurements of HP1 density inside and outside of the droplet allow us to construct the *ρ*-$\varepsilon_{\text{HH}}$ phase diagram for large $\varepsilon_{\text{HC}}$, on which we can also identify the coating regime (Fig. 2 *c* *bottom*, and see supporting material, section 6). There is a further new region on this phase diagram (the shaded stripe) where a droplet forms only due to HP1-chromatin interactions (i.e., $\varepsilon_{\text{HH}}$ is not large enough for a protein droplet to form on its own). We discuss this in more detail below.

To characterize these regimes more quantitatively (and to determine the positions of the lines in Fig. 2 *b*), we measure the local protein density by splitting the simulation box into $N_{\text{sb}}$ sub-boxes of volume $V_{\text{sb}}$. If there are $N_{i}$ HP1s in the *i*th sub-box, the local density is $\rho_{i}=N_{i}/V_{\text{sb}}$. To discriminate between the droplet regimes and the weak phase separation observed in the coating regime, we consider a “separation depth” parameter (37) defined as

(1) $$\varphi_{\text{sep}}=\frac{1}{N_{\text{sb}}}\sum_{i=1}^{N_{\text{sb}}}\frac{\rho_{i}-\rho }{\rho^{\text{∗}}-\rho },$$

where $\rho =N/l_{x}^{3}$ is the overall number density of HP1s, and $\rho^{\text{∗}}$ is a reference density that takes the value $\rho_{+}$ when $\rho_{i}>\rho_{+}/2$ and $\rho_{-}=0$ otherwise. This measures the mean local deviation from the uniform density, normalized by the expected deviation for a strongly phase separated system. We use $\rho_{+}=0.5$ and $N_{\text{sb}}=125$, chosen to be optimal for distinguishing the different regimes and leading to $\varphi_{\text{sep}}\to 1$ on droplet formation. Fig. 2 *d* shows how $\varphi_{\text{sep}}$ varies with $\varepsilon_{\text{HH}}$, for different values of $\varepsilon_{\text{HC}}$. For $\varepsilon_{\text{HC}}\leq 12k_{B}T$ the points sit on top of each other (Fig. 2 *d* *inset*), and we observe a sharp crossover (at $\varepsilon_{\text{HH}}\approx 4.5k_{B}T$) from $\varphi_{\text{sep}}∼0.15$ in the mixed phase to $\varphi_{\text{sep}}∼1$ in the dense droplet phase. As noted above, for these values of the energy the model behaves qualitatively the same as, e.g., interacting Brownian colloids (30), and we expect a first-order phase transition in the thermodynamic limit (model B). We use a value of $\varphi_{\text{sep}}=0.5$ to set the position of the orange line in Fig. 2 *b*. As $\varepsilon_{\text{HC}}$ increases, this line shifts to the left—we discuss this interesting regime further below. Fig. 2 *e* shows that, for small $\varepsilon_{\text{HH}}$, the separation depth is independent of $\varepsilon_{\text{HC}}$ throughout the uniform phase ($\varphi_{\text{sep}}∼0.15$), before increasing at larger $\varepsilon_{\text{HC}}$ in the coating or absorbing droplet regimes; we take the value of $\varepsilon_{\text{HC}}$ at which $\varphi_{\text{sep}}$ starts to increase as the point where the system enters the coating regime (*green dashed line* in Fig. 2 *b*). For $\varepsilon_{\text{HH}}>6k_{B}T$ the separation depth $\varphi_{\text{sep}}∼1$, independently of $\varepsilon_{\text{HC}}$; i.e., this parameter cannot differentiate between droplets and absorbing droplets.

**Figure 3:**
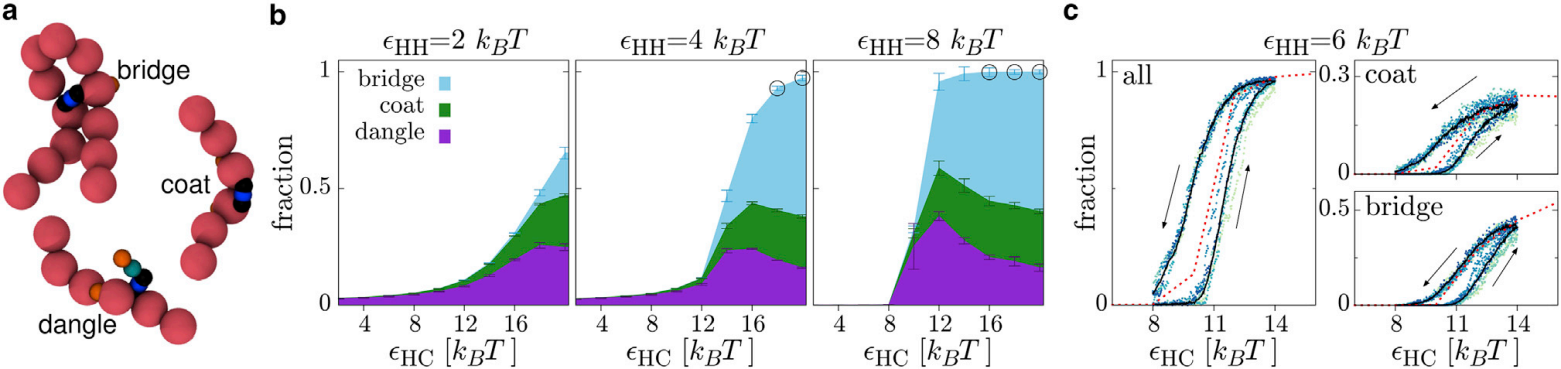
Protein-chromatin binding modes. (*a*) Simulation snapshots of a single model HP1 and a short section of chromatin. The three different binding modes are depicted. (*b*) Plots showing the fraction of the N=1000 proteins bound to the chromatin in each mode for different interaction energies. The height of each colored region indicates the proportion of proteins, with the regions stacked on top of each other. In this way, the height of the total colored region indicates the fraction of proteins bound in any mode $f_{\text{tot}}$. Values are obtained from averaging over four simulations of duration $5\times 10^{3}\tau$, and error bars show the standard error in the mean. Black circles around points indicate where bridging is the dominant binding mode (more than half of the bound proteins). (*c*) Plots showing the fraction of proteins bound (total or in the indicated mode) during a simulation where the HP1-chromatin interaction energy was slowly varied: starting at $\varepsilon_{\text{HC}}=8k_{B}T$ it was increased to $\varepsilon_{\text{HC}}=14k_{B}T$ over $2\times 10^{4}\tau$, before being decreased again over the same time interval. The HP1-HP1 interaction energy was kept fixed at $\varepsilon_{\text{HH}}=6k_{B}T$. Blue-green points show data from 12 independent simulations, each in a different color. Black solid lines show an average over these simulations. The red dotted line shows values obtained from equilibrium simulations as in (*b*). To see this figure in color, go online.

We now consider the nature of the interactions between the HP1 dimers and the chromatin. Since each model HP1 dimer can interact with the polymer via two distinct domains (the CDs), they can bind in three different modes (Fig. 3 *a*). First, an HP1 could bind through only one of the CDs; we call this “dangling,” since it leaves one free CD. Second, the CDs could both bind to the chromatin at adjacent (|i−j|<2) polymer beads; we call this “coating.” Finally, if the CDs interact with polymer beads that are separated along the chain (|i−j|≥2), then the protein is “bridging.” As detailed in (21), the shape of the protein determines its likelihood to bind in each mode: bridging incurs an entropic penalty (due to polymer looping), so unless the shape of the protein specifically disfavors coating, the coating mode is favorable. This is the case here: in the absence of protein-protein interactions we mainly observe coating. The ability of real HP1 dimers to form bridges between distant chromatin regions remains unclear; however, cryoelectron microscopy (38) and detailed molecular simulations (39) have indicated that HP1 can readily sit between adjacent nucleosomes, suggesting that (at least under dilute conditions) coating may well dominate.

In Fig. 3 *b* we plot the fraction of bridging, coating, and dangling proteins as a function of $\varepsilon_{\text{HC}}$. If we consider the total fraction of proteins bound to the polymer $f_{\text{tot}}$ at small $\varepsilon_{\text{HH}}=2k_{B}T$, $f_{\text{tot}}$ increases smoothly with $\varepsilon_{\text{HC}}$. Coating and dangling are the dominant binding modes; the BIA is therefore not in effect, and we do not observe BIPS or chromatin compaction. At large $\varepsilon_{\text{HH}}$, where there is a droplet, $f_{\text{tot}}$ increases very sharply as $\varepsilon_{\text{HC}}$ is increased and the polymer becomes absorbed into the droplet (the curve becomes steeper from left to right in the panels of Fig. 3 *b*). This could indicate the presence of a first-order phase transition in the thermodynamic limit. Within the absorbing droplet regime we also observe that the fraction of bridging proteins increases with $\varepsilon_{\text{HC}}$, and it becomes the dominant mode of binding when both interactions are strong. The main driver of this is that, as $\varepsilon_{\text{HC}}$ increases, more of the polymer becomes absorbed, and so the likelihood of two distant regions being close enough together for bridges to form increases. We also performed some simulations where the HP1-chromatin interaction was increased slowly (after starting in an equilibrium configuration), before being decreased again (Fig. 3 *c*). As detailed further in supporting material, section 7, the system displays hysteresis as the polymer becomes absorbed and then reemerges from the droplet. These observations suggest that, in the limit of a large droplet, the system would show a first-order transition as $\varepsilon_{\text{HC}}$ increases, to a phase where the polymer is fully absorbed; in our small system we instead observe an extended coexistence regime where the polymer is only partially absorbed. We use the point where $f_{\text{tot}}=0.5$ to set the position of the solid green line in Fig. 2 *b*.

**Figure 4:**
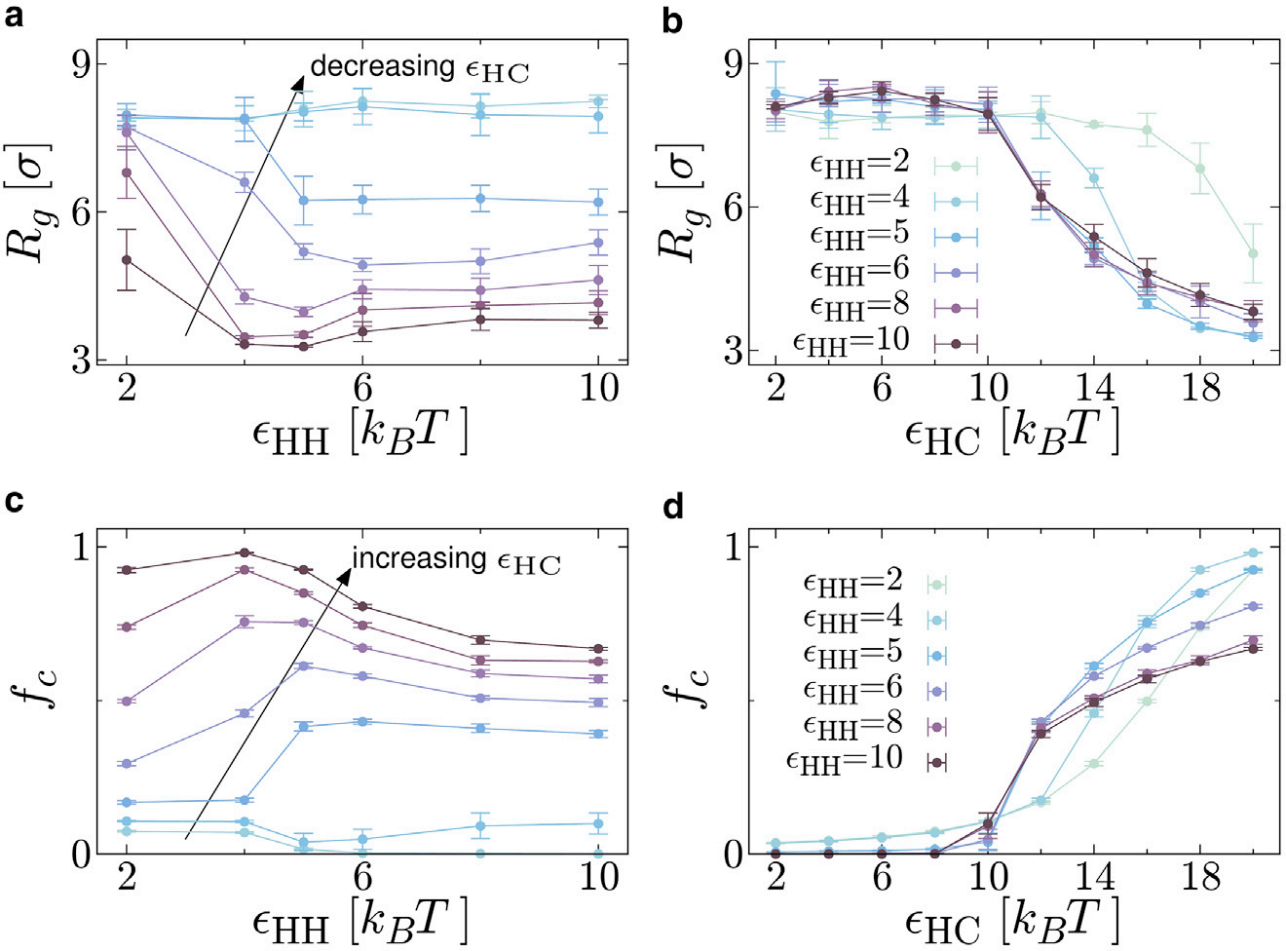
HP1-chromatin interactions and chromatin compaction. (*a* and *b*) Plots showing how the radius of gyration of the polymer representing the chromatin segment depends on the interaction energies. In (*a*) curves are for $\varepsilon_{\text{HC}}$ values between 8 and 20 $k_{B}T$ increasing in steps of 2 $k_{B}T$ from top to bottom. Points are obtained from an average of four independent simulations; error bars show the standard error in the mean, and connecting lines are a guide to the eye. (*c* and *d*) Plots showing how the fraction of chromatin beads that are bound by proteins $f_{c}$ depends on the interaction energies. In (*c*) from bottom to top, curves are for $\varepsilon_{\text{HC}}$ values between 8 and 20 $k_{B}T$, increasing in steps of $2k_{B}T$. Again, points are obtained from an average of four independent simulations; error bars show the standard error in the mean, and connecting lines are a guide to the eye. To see this figure in color, go online.

One proposed function of HP1 in vivo is to compact heterochromatin. The ability of our model proteins to compact the chromatin can be probed by measuring its radius of gyration, defined as

(2) $$R_{g}^{2}=\frac{1}{L}\sum_{i=1}^{L}{\left(r_{i}-\bar{r}\right)}^{2},$$

where $r_{i}$ is the position of the *i*th chromatin bead, and $\bar{r}=\left(1/L\right)\sum_{i}r_{i}$. Fig. 4, *a* and *b* show how $R_{g}$ depends on the interaction energies. Interestingly, $R_{g}$ can vary nonmonotonically as $\varepsilon_{\text{HH}}$ increases; similar behavior is observed in the fraction of polymer beads bound by proteins, $f_{c}$ (Fig. 4, *c* and *d*)). The reason for this nonmonotonicity is strikingly apparent in the top row of snapshots in Fig. 2 *a*: in the leftmost snapshot the polymer is swollen, in the second from the left it is fully absorbed into a protein droplet (small $R_{g}$ and large $f_{c}$), but in the two right-hand snapshots the polymer is only partially absorbed into the droplet ($R_{g}$ increases again, while $f_{c}$ decreases). That the amount of absorbed polymer varies so widely within the absorbing droplet regime is likely due to competition between different contributions to the free energy. While HP1-chromatin binding represents a reduction in free energy, this is offset by the reduction in entropy due to the compaction/confinement of the polymer within the droplet. Increasing $\varepsilon_{\text{HC}}$ increases the amount of chromatin absorbed as the entropic loss is overcome. On the other hand, the presence of the polymer within a droplet will reduce the number of HP1-HP1 interactions due to steric effects; so increasing $\varepsilon_{\text{HH}}$ *decreases* the amount of chromatin absorbed (effectively the polymer is “squeezed out” of the droplet).

Finally in this section, we consider intermediate values of the HP1-HP1 interaction strength, $\varepsilon_{\text{HH}}\approx 4k_{B}T$, where we observe the most interesting behavior. Here, in the absence of chromatin interactions there is no droplet formation and $\varphi_{\text{sep}}$ is small. However, we note that, as $\varepsilon_{\text{HC}}$ increases, the orange line in Fig. 2 *b* moves to the left, so droplets *can* form at $\varepsilon_{\text{HH}}\approx 4k_{B}T$ *if* the protein-chromatin interaction energy is large enough. In other words, HP1-chromatin attraction promotes protein aggregation. This is reminiscent of the scaffold-client systems studied via simulations of multivalent patchy particles (12, 13, 14), but here the presence of a long polymer allows for the BIA to be in effect. The behavior can be understood as follows: when $\varepsilon_{\text{HC}}$ is large enough, a significant number of HP1s become localized to the polymer and these tend to bind in the coating mode. Then, intermediate HP1-HP1 interactions are sufficient to allow extended chromatin-HP1-HP1-chromatin bridges to form. The BIA then leads to chromatin compaction and protein clustering; we note that this is the *only* region of the phase diagram where the BIA is really in effect and a true BIPS is observed. When both $\varepsilon_{\text{HH}}$ and $\varepsilon_{\text{HC}}$ have intermediate values we observe an absorbing (BIPS) protein droplet *and* coating of the chromatin, which emerges from the droplet (cross-hatch shaded region in Fig. 2 *b*).

### Varying protein density

We now consider the effect of the overall protein density for the multivalent HP1 model. As expected, for large $\varepsilon_{\text{HH}}$, we observed the same behavior as a standard (model B) phase separation: increasing overall protein density leads to an increase in the size of the droplet, while the density of proteins within it remains constant. If $\varepsilon_{\text{HC}}$ is also large, the amount of absorbed chromatin grows with the size of the droplet $R_{d}$ (Fig. S8 and see supporting material, section 9). Interestingly, once the droplet is large enough to fully absorb the polymer, further increasing *N* (and further increase of the droplet size) does not lead to a swelling of the polymer: the ratio $R_{g}/R_{d}$ continues to decrease with *N* (Fig. S8 *e*). When $\varepsilon_{\text{HH}}$ is small and $\varepsilon_{\text{HC}}$ is large (coating regime), varying the number of proteins has little qualitative effect on the system. As *N* increases, the total number of proteins bound to chromatin increases and the ratios of binding in the different modes changes slightly; strikingly, this does *not* lead to further polymer compaction (Fig. S9 and see supporting material, section 9).

**Figure 5:**
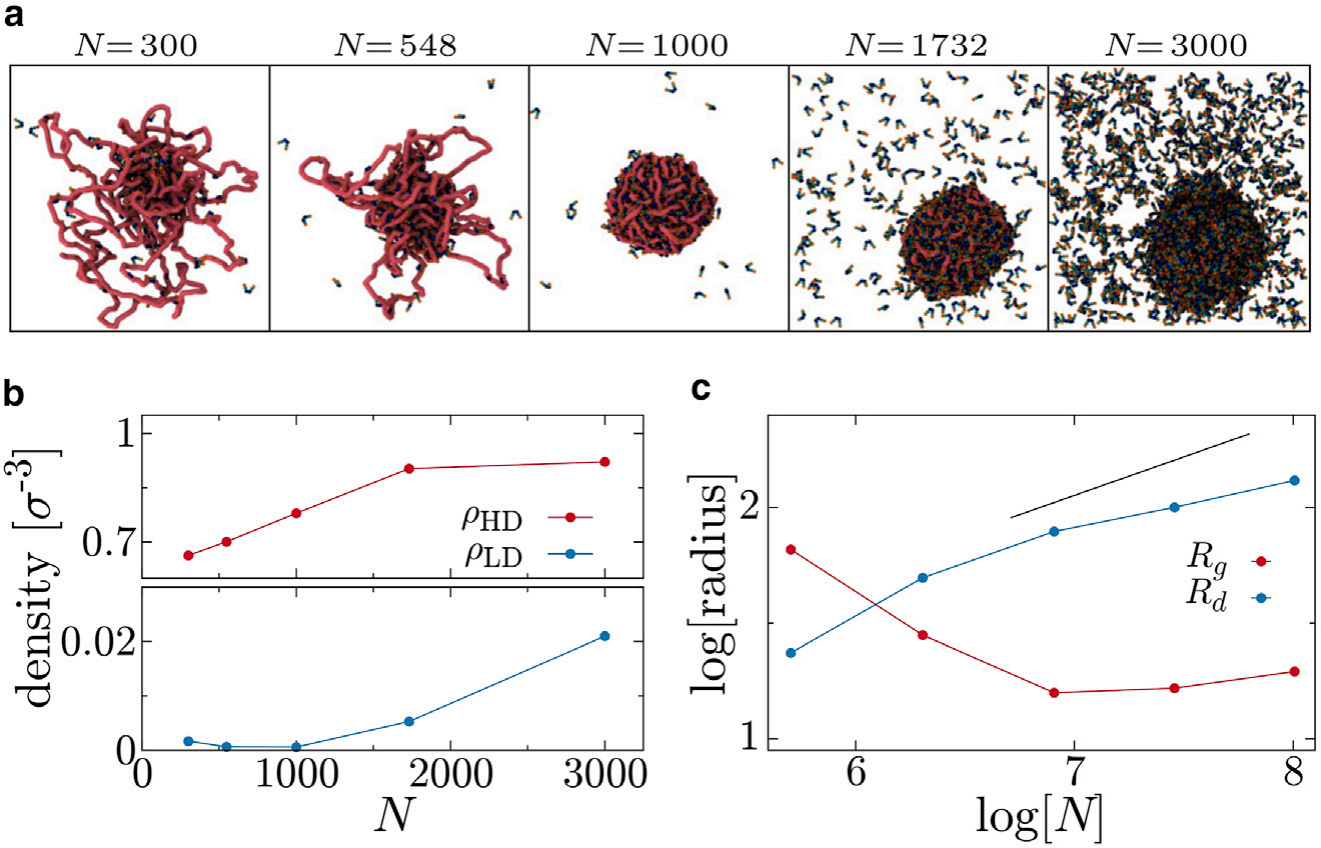
Varying overall protein density. (*a*) Snapshots from simulations with $\varepsilon_{\text{HH}}=4k_{B}T$ and $\varepsilon_{\text{HC}}=20k_{B}T$ but with different numbers of proteins *N* as indicated. (*b*) Plot showing how the protein densities within the high- and low-density phases (inside and outside the droplet) vary with the number of proteins. (*c*) Plot showing how the radius of gyration of the polymer $R_{g}$ and radius of the droplet $R_{d}$ vary with *N*, shown in log-log scale. The black line has a slope 1/3, which is how the droplet radius would scale in a standard phase separating system. To see this figure in color, go online.

A strikingly different behavior is observed for intermediate $\varepsilon_{\text{HH}}$ (the region where the BIA is in effect, i.e., where a droplet only forms due to the presence of the polymer). Fig. 5 *a* shows snapshots for $\varepsilon_{\text{HH}}=4k_{B}T$ and $\varepsilon_{\text{HC}}=20k_{B}T$ with different numbers of proteins. It is immediately clear that the density of proteins within the two phases varies with *N* (also Fig. 5 *b*). This can be rationalized as follows. For small *N* a protein droplet forms on the polymer via the BIA. This droplet is rather “loose” and, as *N* increases, more space within the droplet becomes filled with proteins and the density ($\rho_{\text{HD}}$) increases. At the same time more polymer becomes absorbed and the droplet grows ($R_{g}$ decreases, and the droplet diameter $R_{d}$ increases, Figs. 5 *c* and S10). When N≈1000 all of the polymer is absorbed, and $R_{g}$ reaches a minimum; as *N* and $R_{d}$ increase further the polymer can swell slightly. At some point the droplet density plateaus, and adding further proteins instead leads to an increase in the density of proteins outside the droplet ($\rho_{\text{LD}}$). The droplet still grows with *N*, but more slowly than in a standard phase separation (where $R∼N^{1/3}$); the fraction of proteins binding the polymer in the bridging mode decreases at the expense of the other two modes (Fig. S10 and see supporting material, section 9).

In summary, for the narrow range of parameters where phase separation only occurs in the presence of the polymer, we find the surprising result that the density of the phases ($\rho_{\text{HD}}$ and $\rho_{\text{LD}}$) depends on the overall protein density (*shaded band* in Fig. 2 *d*). This has important implications for protein-chromatin interaction in vivo (see Conclusion).

### Model 2: Limited valence protein-protein interactions

In this model the HP1 dimer-dimer interactions have a limited valence, i.e., exactly one hinge domain can interact with exactly one NTE domain at a time. In the previous section we specifically considered equilibrium configurations; here, we found that, even after very long simulation run times, the limited valence model configurations could still depend on the initial conditions (Fig. S12, *a* and *b*). This is similar to classic low-valence patchy particles, which have been studied extensively using both simulations (28, 29) and experiments (31, 32). Patchy particles have a rich phase diagram which includes a low-density equilibrium gel phase and “closed loop” structures (where a set of particles form a structure where all patches are bound). Technically, these different but coexisting equilibrium states are only present at zero temperature, but the structures can also exist as very long-lived nonequilibrium metastable states for non-zero temperatures (30). Similar behavior has also been observed in sticker-spacer types models (40). Since we could not guarantee that our simulated structures represented the equilibrium state, we instead study the metastable states obtained when the system is quenched by instantaneously switching on both protein-protein and protein-chromatin interactions. Specifically, we start from an equilibrium configuration for $\varepsilon_{\text{HH}},\varepsilon_{\text{HC}}=0$, switch on interactions, and run for $10^{4}\tau$ (where *τ* is the simulation time unit); after this time the measured quantities ($f_{c}$, $\varphi_{\text{sep}}$, etc.) have stopped systematically varying. Steady-state values of these quantities are then obtained by averaging over a further $10^{4}\tau$ simulation.

**Figure 6:**
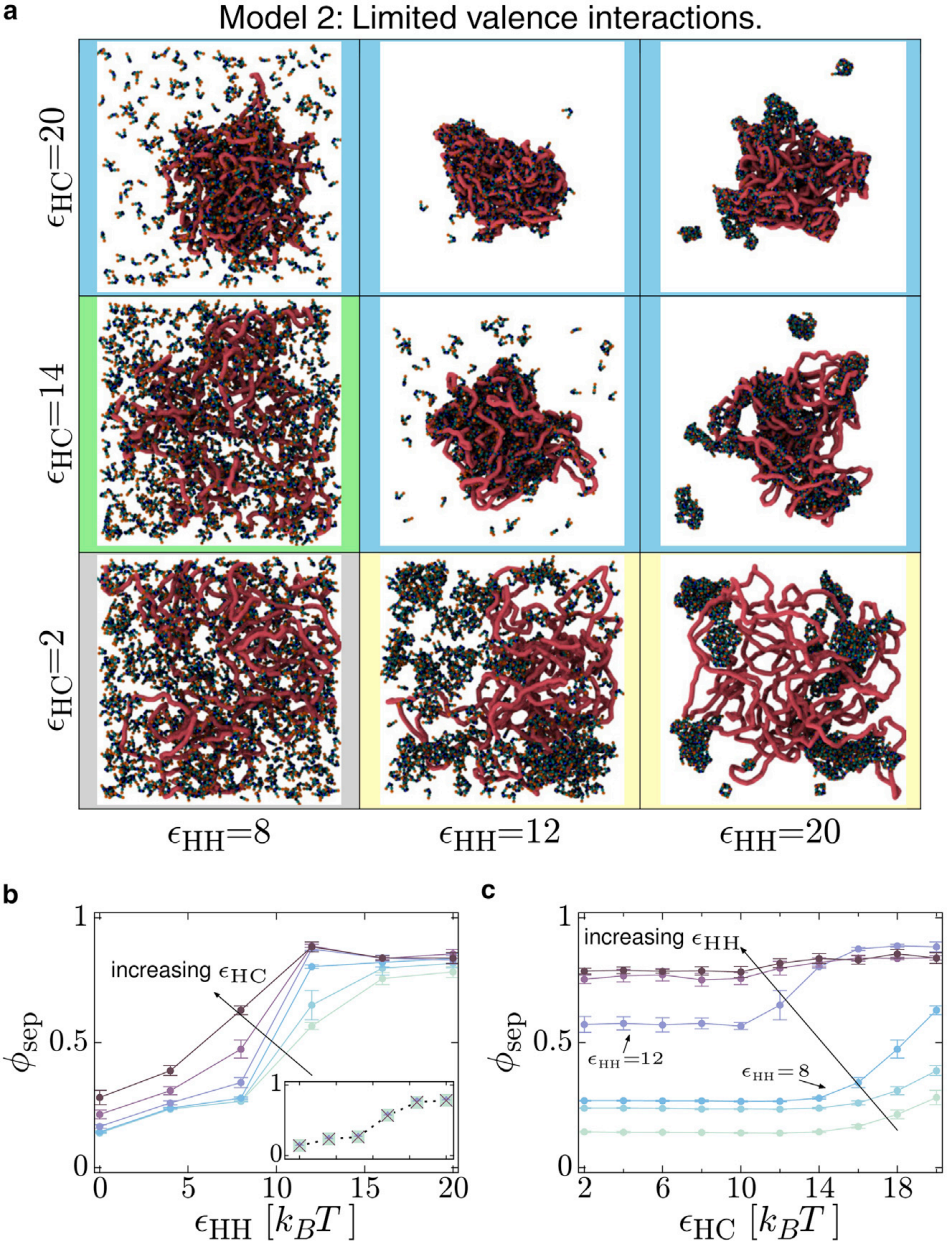
The limited valence HP1s display similar behavior regimes. (*a*) Snapshots are shown for simulations of the limited valence HP1 model with different HP1-HP1 and HP1-chromatin interaction energies. Border colors indicate similarity to the different regimes observed for the multivalent model in Fig. 2 (see also Fig. S13). (*b*) Plot showing how the separation depth parameter varies with $\varepsilon_{\text{HH}}$ for different values of $\varepsilon_{\text{HC}}$ for the limited valence model. Data for $\varepsilon_{\text{HC}}$ between 10 and 20 $k_{B}T$ increasing in steps of 2 $k_{B}T$ are shown in the main plot. The inset shows that points for $\varepsilon_{\text{HC}}$= 6, 8, and 10 $k_{B}T$ sit on top of each other. (*c*) Similar plot showing $\varphi_{\text{sep}}$ as a function of $\varepsilon_{\text{HC}}$. Curves are for different values of $\varepsilon_{\text{HH}}$ between 0 and 20 $k_{B}T$ increasing from bottom to top in steps of 4 $k_{B}T$. To see this figure in color, go online.

Typical snapshots are shown in Fig. 6 *a*. Similar behavior is observed as for the multivalent interaction model. At low $\varepsilon_{\text{HH}}$ we have the same mixed and coating regimes. For low $\varepsilon_{\text{HC}}$, as $\varepsilon_{\text{HH}}$ increases, we go from the mixed phase to an aggregate phase. Unlike the multivalent model, here the aggregates are not spherical; instead multiple irregularly shaped clusters form. We also see small closed clusters of HP1s where all hinge and NTE domains are bonded When both $\varepsilon_{\text{HC}}$ and $\varepsilon_{\text{HH}}$ are large, many of the aggregates become associated with the polymer, which becomes compacted. Some smaller clusters remain detached from the polymer. Measurements of clusters and subclusters (see supporting material, section 10) show that these have a fractal dimension less than 3, as would be expected in a gel. It is important to reiterate that these are long-lived metastable, dynamically arrested structures, and do not represent a true equilibrium of the system. Using a different quenching or annealing procedure with the same parameters leads to different relative abundances of the different types of aggregate (see supporting material, section 11).

As before, we measure the separation depth $\varphi_{\text{sep}}$ as a function of the two interaction energies. Fig. 6 *b* shows that the behavior is again similar to the multivalent model in that $\varphi_{\text{sep}}$ increases with $\varepsilon_{\text{HH}}$. However, the largest $\varphi_{\text{sep}}$ values are smaller than in the multivalent case, consistent with several protein aggregates of different size forming, rather than a single phase separated droplet. There is also a regime where proteins aggregate only when the interaction with the chromatin is strong enough, although it is less clear than for the multivalent model. Specifically, for $\varepsilon_{\text{HH}}=8k_{B}T$ there is a cluster only when $\varepsilon_{\text{HC}}$ is large, but $\varphi_{\text{sep}}$ only reaches intermediate values (Fig. 6, *a* and *c*). For $\varepsilon_{\text{HH}}=12k_{B}T$, $\varphi_{\text{sep}}$ has an intermediate value just less than 0.6 for a broad range of $\varepsilon_{\text{HC}}$ values (Fig. 6 *c*), behavior that is not observed in the multivalent model. This arises because, while clusters do form, there are many of them; they are also highly dynamic, continually forming, dissolving, and merging and breaking apart (30).

We again measure the fraction of proteins bound in different modes (Fig. S14), the fraction of polymer beads bound by proteins (Fig. S15, *a* and *b*), and the radius of gyration of the polymer (Fig. S15, *c* and *d*)) as a function of the two interaction energies. The behavior is broadly similar to the multivalent model, but the limited valence proteins are less able to compact the polymer, and the chromatin “looping out” (which was observed in the multivalent case when both energies were large) does not tend to occur here. Instead, most of the polymer is associated with the irregularly shaped cluster.

### Protein dynamics

**Figure 7:**
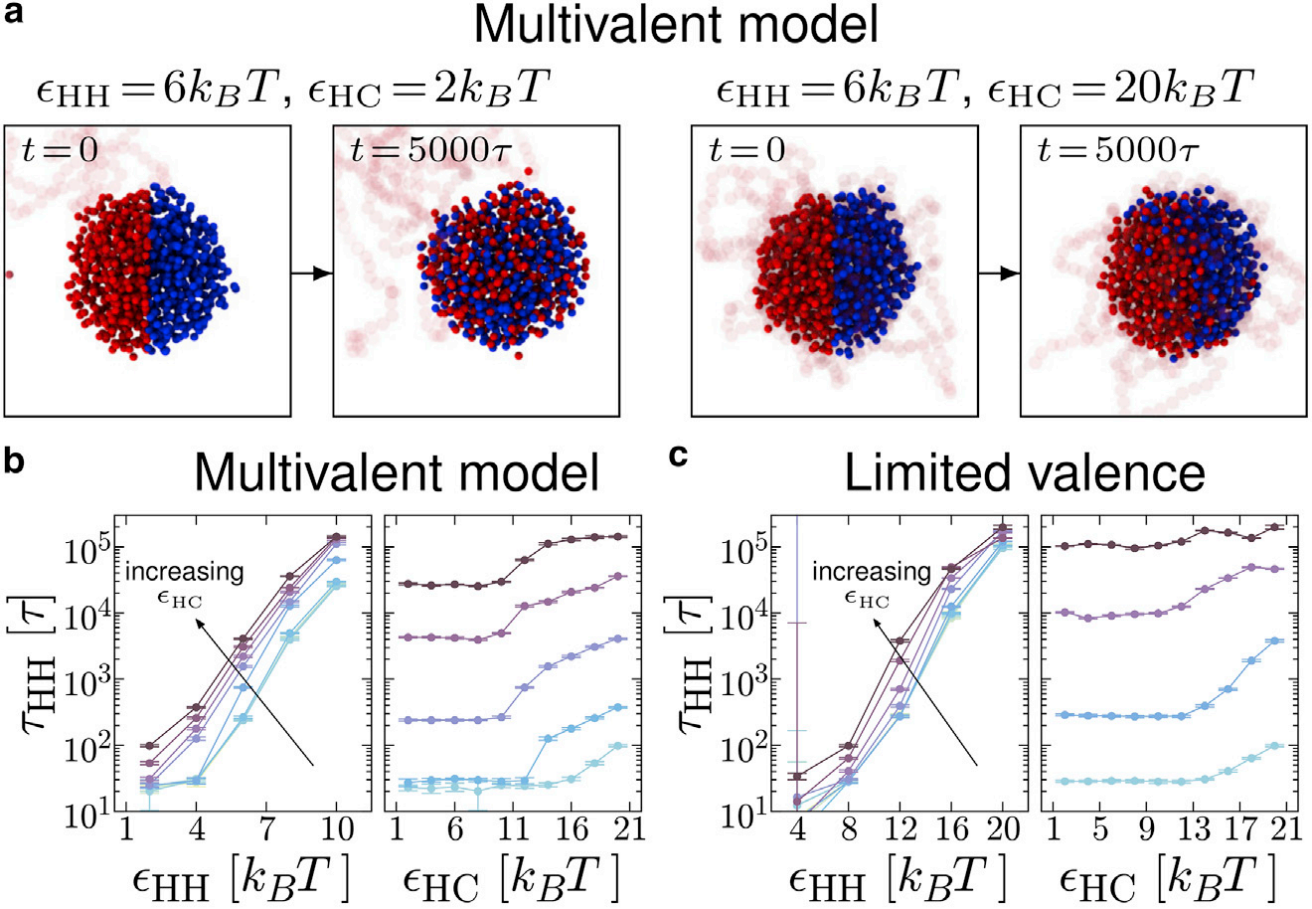
Dynamics of protein-protein interactions. (*a*) Simulation snapshots showing protein mixing over time for two different parameter sets for the multivalent HP1 model. At t=0 we color each protein according to which half of a droplet it is in; for simplicity only one bead from each protein is shown, and the polymer is shown transparent. Images at t=0 and $t=5\times 10^{3}\tau$ are shown (this corresponds to an interval of the order 3–4 min). For $\varepsilon_{\text{HH}}=6k_{B}T$, $\varepsilon_{\text{HC}}=2k_{B}T$ (*left*) the system is in the droplet phase and the proteins are not associated with the chromatin, while $\varepsilon_{\text{HH}}=6k_{B}T$, $\varepsilon_{\text{HC}}=20k_{B}T$ (*right*) is in the absorbing droplet regime. (*b*) Plots for the multivalent model showing the HP1 bond decorrelation time constant $\tau_{\text{HH}}$ in simulation time units *τ* as a function of the HP1-HP1 and HP1-chromatin interaction energies. Points show $\tau_{\text{HH}}$ values obtained from fits as detailed in supporting material, section 12 and Fig. S16. Error bars show the error obtained from the fit and connecting lines are a guide for the eye. In the left plot from bottom to top (light to dark colors) lines are for increasing $\varepsilon_{\text{HC}}$ (between 2 and 20 $k_{B}T$ in steps of 2 $k_{B}T$), while in the right plot bottom to top shows increasing $\varepsilon_{\text{HH}}$ (between 2 and 10 $k_{B}T$ in steps of 2 $k_{B}T$). (*c*) Similar plots are shown for simulations with the limited valence model. In the left plot lines are for $\varepsilon_{\text{HC}}$ values between 2 and 20 $k_{B}T$ in steps of 2 $k_{B}T$; in the right plot lines are for $\varepsilon_{\text{HH}}$ between 8 and 20 $k_{B}T$ in steps of 4 $k_{B}T$. Points for $\varepsilon_{\text{HH}}=4k_{B}T$ are not shown since the data were noisy and the fitted $\tau_{\text{HH}}$ values had very large errors (see supporting material). To see this figure in color, go online.

So far we have considered structural properties of the protein clusters for each of the two models. Here, we consider protein dynamics. This is often studied in vivo using fluorescence recovery after photobleaching (FRAP) experiments: the timescale of fluorescence recovery of a protein droplet gives a measure of how quickly proteins are exchanged between the droplet and the soluble (unbleached) pool. The internal dynamics of a droplet can also be probed by photobleaching half of the droplet: tracking fluorescence in the bleached and unbleached halves gives information on the relative timescales of mixing within the droplet and exchange with the soluble pool (41). A similar effect can be observed visually in simulations by coloring proteins according to which half of a droplet they are in, and then watching how the colors mix in time. Two examples for the multivalent proteins (model 1) with different $\varepsilon_{\text{HC}}$ values are shown in Fig. 7 *a*. We observe that, for small $\varepsilon_{\text{HC}}=2k_{B}T$ (where the droplet is not associated with chromatin), there is a high degree of mixing over the duration of the simulation. Interestingly, for larger $\varepsilon_{\text{HC}}=20k_{B}T$ (but the same value of $\varepsilon_{\text{HH}}=6k_{B}T$) where the chromatin is absorbed into the droplet we find that the colors mix to a much lesser extent.

More quantitatively, we can measure how the proteins change their binding partners during a given time interval, Δt, by defining a bond-bond correlation function

(3) $$\nu_{\text{HH}}\left(\text{Δ}t\right)=\frac{{\left\langle y_{ij}\left(t+\text{Δ}t\right)\;y_{ij}\left(t\right)\rangle -\langle y_{ij}\left(t\right)\right\rangle }^{2}}{{\left\langle y_{ij}{\left(t\right)}^{2}\rangle -\langle y_{ij}\left(t\right)\right\rangle }^{2}},$$

where $y_{ij}\left(t\right)$ has a value of 1 if proteins *i* and *j* are interacting at time *t*, and 0 otherwise (proteins are said to interact if a hinge or NTE domain on protein *i* is within the interaction range from an NTE or hinge on protein *j*). Angle brackets denote an average over time *t*, repeat simulations, and all possible i,j pairs of proteins. As detailed in supporting material, the shapes of the $\nu_{\text{HH}}\left(\text{Δ}t\right)$ curves suggest that there are multiple timescales involved in the decorrelation; it is nevertheless possible to extract a single overall decorrelation time, $\tau_{\text{HH}}$ (the typical time for all proteins to change their binding partners, see supporting material, section 12 and Fig. S16 for details).

In Fig. 7 *b* we plot $\tau_{\text{HH}}$ as a function of the different interaction energies for the multivalent model; $\tau_{\text{HH}}$ grows roughly exponentially with $\varepsilon_{\text{HH}}$ (linearly on the log-linear plot; this occurs even at high $\varepsilon_{\text{HH}}$). More interestingly, as $\varepsilon_{\text{HC}}$ is increased (Fig. 7 *b* *left*) there is a clear step change in $\tau_{\text{HH}}$ where the system goes from the droplet to the absorbing droplet regime. In other words, consistent with the snapshots in Fig. 7 *a*, the presence of chromatin within the droplet leads to a dramatic slow-down of protein dynamics ($\tau_{\text{HH}}$ increases by almost an order of magnitude). As $\varepsilon_{\text{HC}}$ is further increased there is again a roughly exponential dependence of $\tau_{\text{HH}}$ on $\varepsilon_{\text{HC}}$ (linear increases for $\varepsilon_{\text{HC}}≳12k_{B}T$ on the log-linear scale in Fig. 7 *b* *left*).

There are two main physical reasons for the slow-down upon polymer absorption. First, since polymer bead motion is constrained by their connection to neighboring beads in the chain, the motion of proteins bound to these beads becomes similarly constrained (observing that faster protein dynamics is restored if polymer bonds are “cut” confirms this). Second, if protein-chromatin bonds are effectively stronger than protein-protein bonds, this will also lead to a step-like slow down when the polymer becomes absorbed.

The limited valence proteins (Fig. 7 *c*) show similar behavior. The decorrelation time $\tau_{\text{HH}}$ again grows roughly exponentially with $\varepsilon_{\text{HH}}$ (although there is some deviation from this for large $\varepsilon_{\text{HC}}$). Fig. 7 *c* (*right*) shows that again $\tau_{\text{HH}}$ increases with $\varepsilon_{\text{HC}}$ once the proteins become associated with the polymer; a clear step change is, however, only observed at intermediate values of $\varepsilon_{\text{HH}}$, and this is less pronounced than the multivalent case. This is likely due to the presence of protein clusters that do not interact with the polymer. For larger $\varepsilon_{\text{HH}}$, interaction with the polymer has a much smaller effect, as protein-protein bonds are already longer lived than protein-polymer bonds.

## Conclusion

In this paper we have studied the behavior of simple model proteins interacting with a bead-and-spring polymer model for chromatin. We considered rigid bodies composed of spheres that represent different protein domains that interact attractively with each other or with chromatin. The domain structure was based on that of HP1, but our goal was to obtain insight on the interplay between protein-protein and protein-chromatin interactions in general.

HP1 has been shown to undergo LLPS in vitro (22, 23). That result led to the suggestion that LLPS could play a major role in formation of chromatin-associated protein foci in vivo. Here, we also considered that many protein complexes bind chromatin multivalently, and so the BIA can also play a role. Using a model with multivalent protein-protein interactions, we found that, in the absence of protein-chromatin interactions, increasing the protein-protein interaction strength $\varepsilon_{\text{HH}}$ led to liquid droplet formation (model B). Increasing protein-chromatin attractive interactions led to a sharp crossover to a regime where the chromatin is absorbed into the droplet (with indications that there is a first-order phase transition in the thermodynamic limit). Importantly, the level of chromatin absorption depended on both interaction energies, and the number of proteins/size of droplet. For most of the parameters studied, a significant fraction of the chromatin “looped out” from the droplet (the looping statistics of a similar situation have been studied in (42)). The region of the phase diagram where the chromatin is fully absorbed is narrow, which suggests that precise parameter tuning would be required for protein-protein attraction (LLPS) alone to mediate chromatin-associated protein droplet formation and chromatin compaction/isolation in vivo. On the other hand, the variety of behaviors we observe seems to be robust to changes in details, such as the polymer stiffness or exact shape of the protein model (see supporting material, section 13). Here, we have considered the case where proteins bind nonspecifically anywhere to the chromatin, i.e., simulating a fragment of H3K9me3-modified chromatin; in the future it would also be interesting to consider chromatin with different patterns of binding and nonbinding regions. We would expect that regions where the proteins bind would act as droplet nucleation points; interesting behavior might arise when such nucleation points are kept spatially separated due to nuclear structure, or where they change in time (e.g., if histone modifications change dynamically as genes are activated).

An interesting regime in our multivalent protein simulations is for intermediate values of $\varepsilon_{\text{HH}}$, where a droplet only forms if $\varepsilon_{\text{HC}}$ is large enough. In other words, phase separation is promoted by interaction with chromatin; this can be viewed as chromatin-HP1-HP1-chromatin bridges enabling the BIA to drive protein clustering. In this regime we also see a dependence on the overall protein density *ρ*, which is fundamentally different to standard model B phase separation. The density of proteins within and outside the droplet depends on *ρ*, and the droplet volume grows sublinearly as *ρ* increases. This behavior originates from the formation of a loose protein cluster on the chromatin for small *ρ*, which can “fill up” as proteins are added to the system; at larger *ρ*, sites on the chromatin become saturated; so, as more proteins are added these instead remain unbound (increasing the density in the protein poor region). This is reminiscent of recent work showing that varying the overall concentration of the nucleophosmin protein (a key component of nucleoli, which form via LLPS) leads to variation in its density both inside and outside the nucleolus (43); in that system there are multiple phase separating components, which leads to a complicated high-dimensional phase diagram (see also (12, 13)).

To understand how the behaviors observed in our simulations might play a role in vivo, it is useful to discuss how model parameters map to physical quantities. For the attraction strengths, $\varepsilon_{\text{HH}}$ and $\varepsilon_{\text{HC}}$, it is instructive to consider the “residence times” of the arising interactions. As detailed in supporting material, section 4 (Fig. S2), calibration simulations with single proteins and a short polymer allowed these times to be measured in the absence of cooperative effects. For the multivalent HP1 model, the range of $\varepsilon_{\text{HH}}$ values studied corresponds to interactions residence times ranging from zero to 1 ms (droplets formed for energies equivalent to residence times larger than ∼ 0.7 ms). The protein-chromatin energies studied (which are the same for both models) correspond to residence times of up to 50 ms, with chromatin becoming absorbed into droplets for residence times longer than ∼1 ms. For comparison, experimental measurements of an HP1 dimer interacting with immobilized H3K9me3 chromatin in vitro have shown residence times of 190–250 ms (44); while the mapping for our simulations is only approximate, it is of the correct order of magnitude. The protein concentrations for the simulations presented in Figs. 2 and 6 (where N=1000) map roughly to 39 mM; this is larger than estimates for HP1 in vivo, which are typically in the micromolar range (45). In any case, in most regimes the behavior is independent of overall protein concentration (and in the regime where this is not the case, the ratio of protein to H3K9me3 chromatin is more important than protein concentration per se).

Whether HP1 undergoes LLPS in vivo is still a topic of debate, and there are conflicting observations (41, 46). One recent study showed that overexpression of HP1 in mouse *does not* lead to an increase in the size of foci, but instead the protein density within the foci increases (41). While inconsistent with a classic phase separation mechanism, this observation is compatible with the intermediate $\varepsilon_{\text{HH}}$ (BIA) regime discussed above. The same work used FRAP experiments to show that mixing within HP1 droplets is slower than exchange with the soluble pool (41). Our simulations showed nearly an order of magnitude slow down in protein dynamics when the chromatin is absorbed into the droplet. Due to the small system size, the rate of exchange between the droplet and pool is difficult to measure in our simulations; however, the timescales for exchange with the pool and internal mixing are likely similar. The concurrent slow internal mixing and fast exchange with the pool (of the order 10 s (47)) is therefore not reproduced in the simulations (although see below). Nevertheless, our results suggest that care should be taken when interpreting FRAP measurements of internal mixing: slow mixing may be due to the presence of chromatin, and does not necessarily preclude LLPS. Erdel et al. (41) also showed that removal of the H3K9me3 histone modification leads to loss of HP1 colocalization, but the heterochromatin foci remain intact (inconsistent with HP1 being a driver of heterochromatin body formation). Other work (48) has suggested that, while HP1 may not be necessary to compact large satellite repeat heterochromatin regions, it *is* required to compact and silence smaller H3K9me3 marked segments within otherwise active regions. The function of HP1 is clearly still not well understood. Nevertheless, the simplicity of our model means that our results are also likely to be relevant for other proteins or complexes. An example is the H1 linker histone, associated with chromatin compaction and gene repression; it has been shown to phase separate in the presence of DNA (49), has multiple DNA binding sites, and interacts with the core histones (50).

The limited valence model showed similar regimes to the multivalent case but, instead of a spherical droplet, the proteins formed fractal clusters (similar to the structures formed by patchy particles (28, 29)). The limited valence HP1s could also form a gel in simulations with a higher density and periodic boundaries (Fig. S12). NMR spectroscopy experiments have shown that phosphorylated HP1α forms a gel in vitro if condensates are left for around 7 days (51). A possible explanation for this is that, at first, weak multivalent interactions between the disordered domains drive LLPS (10), with gelation occurring on longer timescales as these rearrange or fold (52). This is consistent with our result that small changes to the nature of the protein-protein interactions can lead to large morphological differences in the resulting condensates (droplets versus fractal clusters), and could be related to the observation that HP1 foci can have quite different properties in different cell types (53). There are also broader implications: LLPS and gelation have been associated with amyloid formation in neurodegenerative disease (54), and it is possible that formation of gels or fractal clusters of chromatin-associated proteins may also be pathological. For example, small disease-associated mutations in the protein MeCP2 (also associated with heterochromatin) were recently shown to prevent it from undergoing LLPS (55).

While above we have highlighted clear similarities between our simulations and previous experimental observations, we hope our results will prompt new experiments to mechanistically test whether these regimes are realizable for HP1 and other proteins. For example, one might consider an in vitro setup with purified protein and long reconstituted chromatin fibers where concentrations could be precisely controlled. Then, spatially varying density and dynamical properties could be measured via fluorescence correlation spectroscopy (FCS), FRAP, or microrheology (56). Or, to probe condensates in vivo, optical droplet technologies could be used to drive protein droplet formation on and off chromatin (57, 58), and then FCS used to probe their properties.

Finally, we note that our model proteins are “poor bridgers,” which tend to coat the chromatin (21). It would be interesting in the future to study the phase diagram of good bridgers (e.g., simple spheres). We have also shown previously that cluster formation via the BIA can be dramatically altered by nonequilibrium chemical reactions that stochastically switch the proteins back and forward between a binding and nonbinding state (modeling posttranslational modifications (19)). Without switching, BIA clusters coarsen and merge until there is a single cluster. Switching arrests coarsening, leading to multiple small clusters where the constituent proteins exchange with the soluble pool at a rate determined by the switching rate. Active reactions have similarly been shown to arrest coarsening in LLPS (59). Such nonequilibrium processes could provide the cell with a means to control droplet formation and size, which does not require precise parameter tuning, allowing fast exchange with a soluble pool but slow internal mixing. It would therefore be of interest in the future to study the interplay between the BIPS and LLPS in a nonequilibrium context.

## Data availability

All simulation and figure data arising from this work are available via the Edinburgh DataShare repository at https://doi.org/10.7488/ds/3474.

## Author contributions

M.A. and C.A.B. performed and designed the research and wrote the paper.

## Supporting citations

References (60, 61, 62, 63, 64, 65, 66, 67, 68, 69, 70) appear in the supporting material.

## Supporting material

Document S1. Figures S1–S18

Document S2. Article plus supporting material
