# Supplementary material for: Simulating the chromatin-mediated phase separation of model proteins with multiple domains
Source: Biophys J. 2022 May 28;121(13):2600–12. doi: 10.1016/j.bpj.2022.05.039 (PMC9300671; doi:10.1016/j.bpj.2022.05.039)
Supplement: Document S1. Figures S1–S18 [file mmc1.pdf]

Biophysical Journal, Volume *121*

Supplemental information

Simulating the chromatin-mediated phase separation of model proteins with multiple domains

Marco Ancona and Chris A. Brackley

Simulating the chromatin mediated phase separation of model proteins with multiple domains

Marco Ancona[1] and Chris A. Brackley[1]

[1]SUPA, School of Physics and Astronomy, University of Edinburgh, Peter Guthrie Tait Road, Edinburgh EH9 3FD, United Kingdom

Supporting Material

Supporting Figures

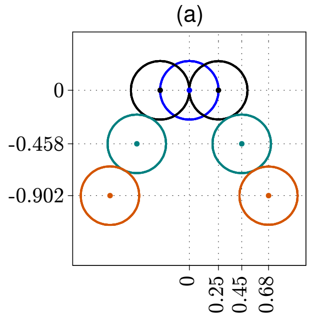

(b)

domain (units)	x (σ)	y (σ)	z (σ)
CSD	0.0	0.0	0.0
hinge (×2)	±0.25	0.0	0.0
CD (×2)	±0.45	−0.458	0.0
NTE (×2)	±0.68	−0.902	0.0

FIGURE S1. Model HP1 rigid body structure. (a) Diagram showing the relative positions of the 7 component beads of the model HP1 dimer. A bead positioned at the origin represents the chromoshadow domain (CSD, blue), which is the dimerisation domain. Other beads represent the two copies (one per monomer) of the hinge domain (black), the chromodomain (green) and the N-terminal end (orange). The same colour scheme is used in Fig. 1 in the main text; units are chromatin bead diameters σ, and all HP1 beads have diameter 0.5σ. (b) Coordinates relative to the CSD.

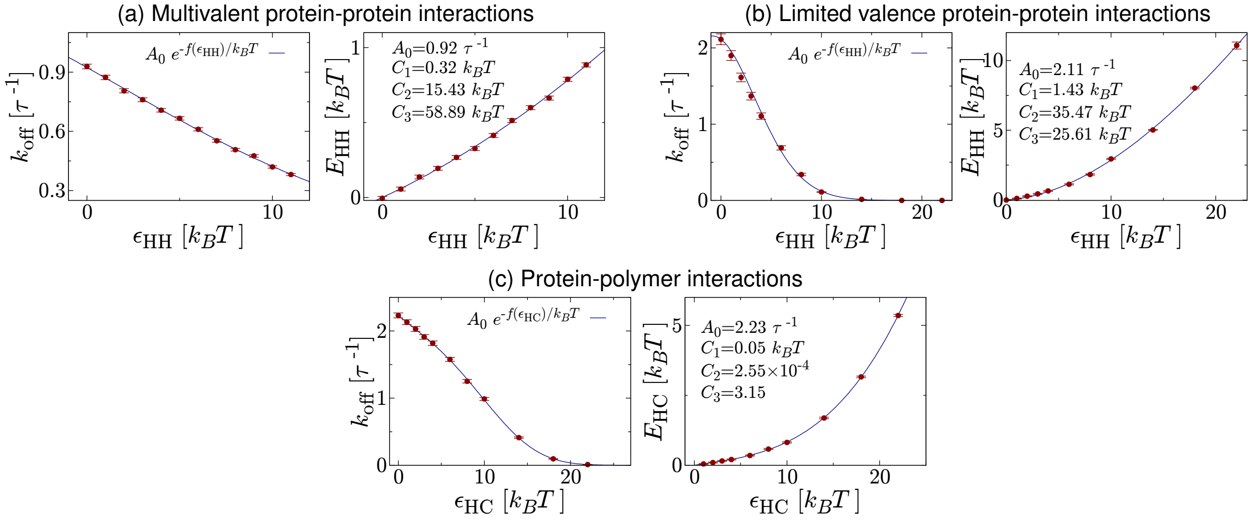

FIGURE S2. Interaction energy calibration. Left-hand plots show the mean duration of binding events as a function of the bare interaction energies ϵ_{HH} or ϵ_{HC} obtained from calibration simulations as detailed in Supporting text, section 4. Points show an average over at least 1000 binding events, and error bars show the standard error in the mean. Lines are obtained from a fit to the data as detailed in Supporting text, section 4. Right-hand plots show the mapping between the effective (E) and bare (ϵ) interaction energies obtained from this fit, with the values of the fit parameters indicated. Panels (a) and (b) show results for protein-protein interactions from the multivalent and limited valence HP1 models respectively. Panel (c) shows results for HP1-chromatin interactions.

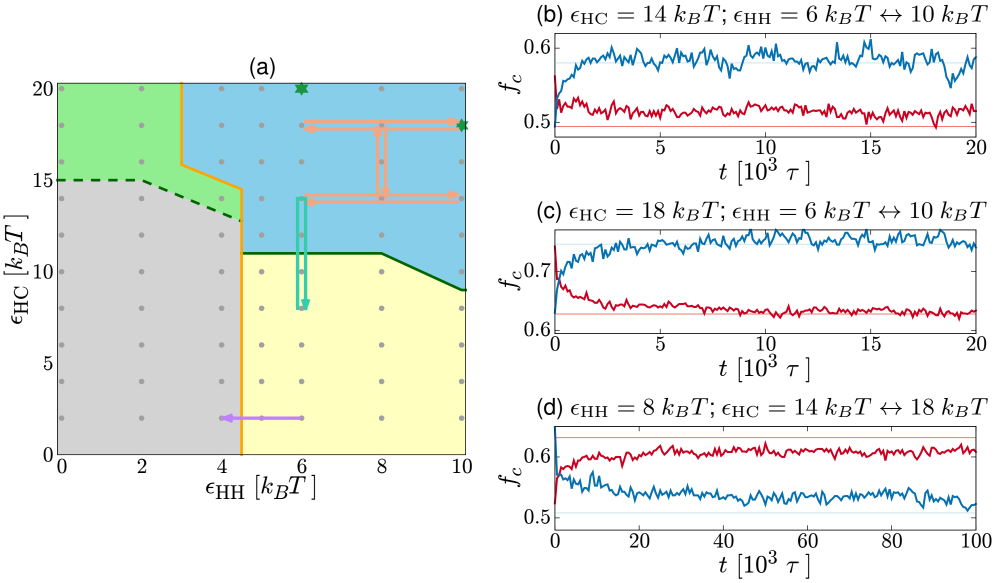

FIGURE S3. Simulated configurations are representative of equilibrium. (a) Phase diagram for multivalent model. Grey points show parameter values for simulations used in Figs. 2-4 in the main text. Peach arrows show quench simulations where, after obtaining an equilibrium configuration for one set of parameters, the energy values were instantaneously changed. The dark-green stars indicate points where replica exchange simulations were performed (see Supporting text, section 5). For comparison, the light-green arrow shows the parameter values used in the hysteresis simulations detailed in Supporting text, section 7 (also Fig. 3(c) in the main text). The purple arrow indicates parameters for a simulation where ϵ_{HC} was instantaneously reduced from $6k_BT$ to $4k_BT$, and we observed that the droplet dissolves (see Supporting text, section 5). On the right-hand plots we show the number of chromatin beads bound to HP1s, f_c, as a function of time after a quench, where the energy values are changed instantaneously. In (b), $\epsilon_{\mathrm{HC}} = 14k_BT$ and ϵ_{HH} is changed from $6k_BT$ to $10k_BT$ at $t = 0$ (dark-red line), or from $10k_BT$ to $6k_BT$ at $t = 0$ (dark-blue line). Pale coloured lines indicate the value of f_c at equilibrium. Panels (c) and (d) show similar plots as indicated.

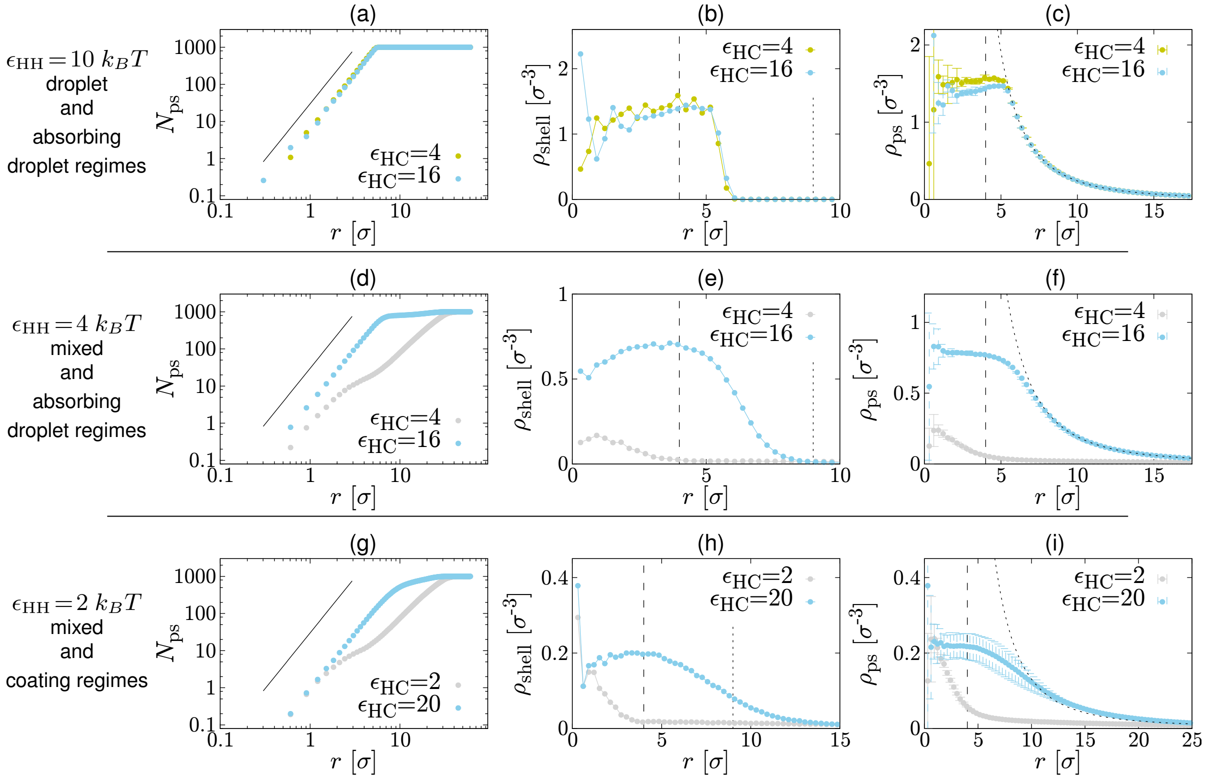

FIGURE S4. Protein densities within and outside protein droplets for the multivalent HP1 model. Each row shows plots obtained for a different value of $\epsilon_{\rm HH}$ with the relevant regimes indicated on the left. (a) Plot showing how the number of proteins $N_{\rm ps}$ within a probing sphere varies with the sphere radius r (log-log scale). The probing sphere is centred on the centre of mass of the largest protein cluster in the system. Results for two different values of $\epsilon_{\rm HC}$ are shown as indicated (units are k_BT). Black lines shows $N_{\rm ps} \sim r^3$, the expected growth for a uniform protein droplet. (b) The density of proteins within a spherical shell of width $dr = 0.3\sigma$ is plotted as a function of r. The abrupt drop-off can be used to extract an estimate of the droplet radius. The dashed and dotted vertical lines correspond to $r_{\rm in}$ and $r_{\rm out}$, respectively (see Supporting text, section 6). (c) Here, the mean density within the entire probing sphere is plotted as a function of r. We estimate the droplet density $\rho_{\rm HD}$ by fitting a horizontal line to the region between σ and $r_{\rm in}$. The black dotted curve represents the expected decay of the density $\rho_{\rm ps} \sim r^{-3}$ outside the droplet. (d-f) Similar plots but for $\epsilon_{\rm HH} = 4k_BT$. Our probing sphere procedure can be applied even in the mixed phase, but it does not make sense to extract a density in this case. For the $\epsilon_{\rm HC} = 16k_BT$ case in the absorbing droplet regime there is a slower drop-off in $\rho_{\rm shell}$ than in panel (c), indicating a broader boundary region (where proteins transiently coat polymer segments which extend out of the droplet). Panels (g-i) show similar plots for $\epsilon_{\rm HH} = 2k_BT$.

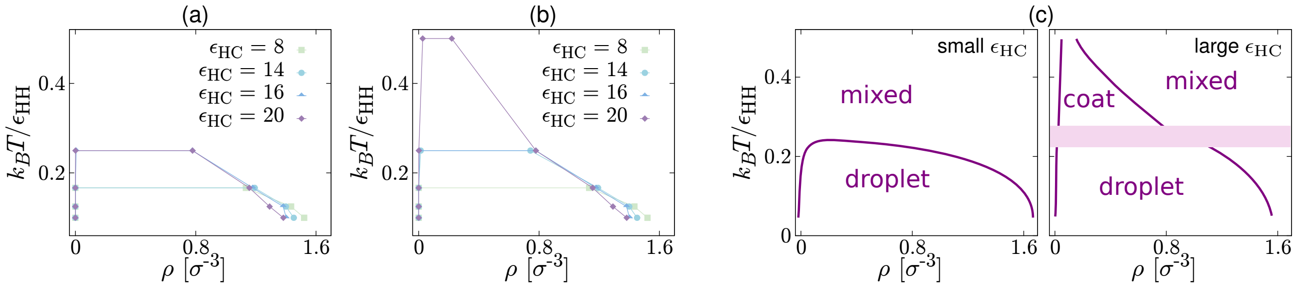

FIGURE S5. Phase densities $\rho_{\rm LD}$ and $\rho_{\rm HD}$ for the multivalent HP1 model. (a) Plot showing values for the protein densities in the low and high density phases, on the ρ-$k_BT/\epsilon_{\rm HH}$ plane. Colour indicates the value of $\epsilon_{\rm HC}$ as indicated; points are only shown for parameter values where $\phi_{\rm sep} > 0.6$. Connecting lines are shown as a guide to the eye, and give approximate boundaries between mixed and droplet regimes. (b) Similar plot, but now points are shown for all parameter values where $\phi_{\rm sep} > 0.2$. This means that there are additional points compared to panel a for large $\epsilon_{\rm HC}$ values; the lines locate the approximate boundaries between mixed and droplet/coating regimes. (c) Sketch phase diagrams for low (left) and high (right) values of $\epsilon_{\rm HC}$ as interpreted from the data in panels (a) and (b).

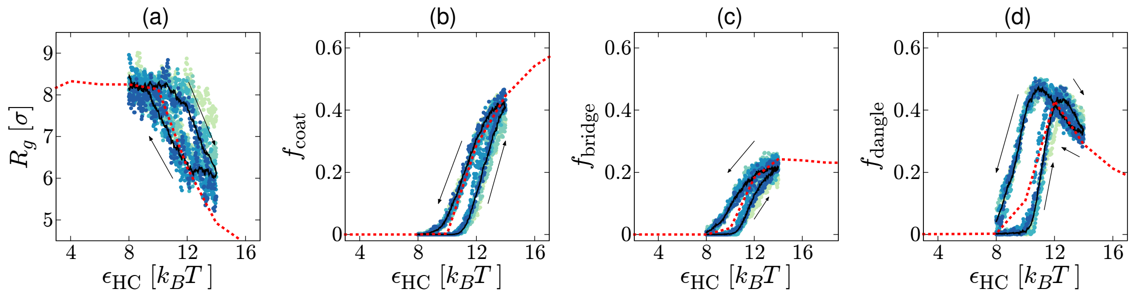

FIGURE S6. Hysteresis in the 'droplet'–'absorbing droplet' transition. (a) Plot showing the polymer radius of gyration obtained from simulations where $\epsilon_{\rm HC}$ is slowly increased from $8k_BT$ to $14k_BT$ before being decreased again as detailed in Supporting text, section 7. Points show values obtained from 12 individual simulations, with each simulation shown in a different colour. The black line shows an average over these repeat simulations, and arrows indicate the direction of time. The red dotted line shows the equilibrium curve [as in Fig. 4(d) in the main text]. (b-d) Similar plot but showing the fraction (of the $N = 1000$ proteins) which are bound to the polymer in each of the different binding modes.

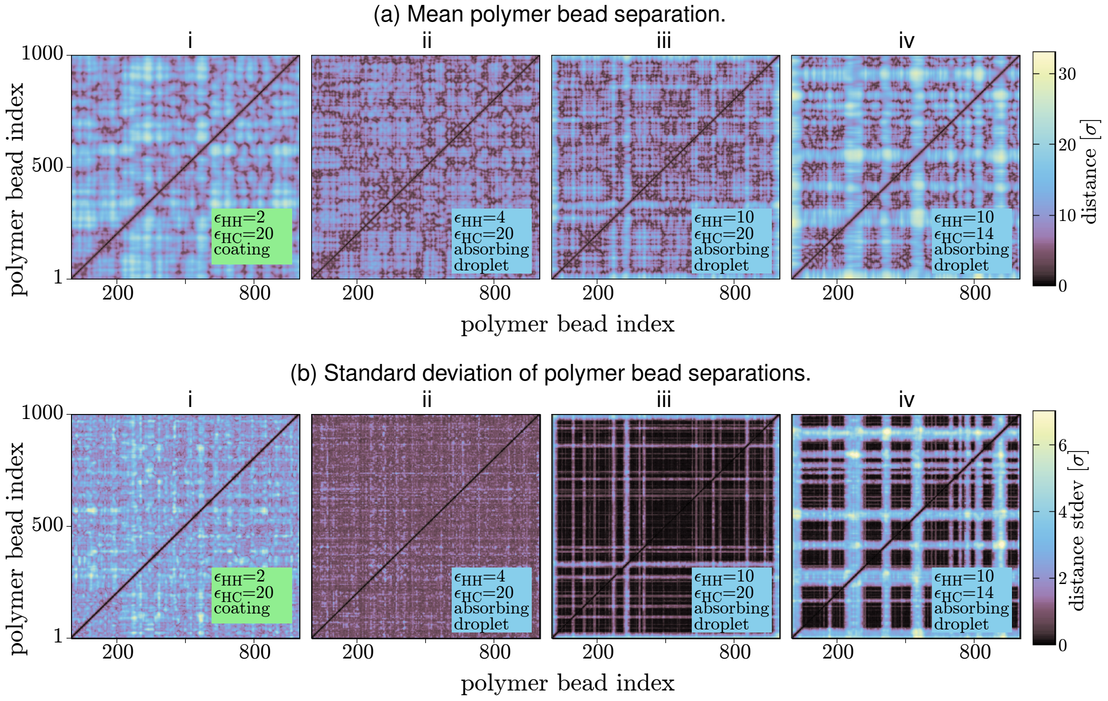

FIGURE S7. Polymer 'distance maps' for the multivalent protein model. (a) Colour maps showing distances between chromatin beads obtained from a time average of single equilibrium simulation (of duration $5 \times 10^3\tau$). The different sub-panels show maps from the different regimes with $\epsilon_{\rm HH}$ and $\epsilon_{\rm HC}$ as indicated (units of k_BT). Distant pairs of chromatin beads (swollen polymer) are shown by brighter colours, while pairs of beads which are close together in 3D space by darker colours (compacted polymer). For example, in map iv, dark and light stripes indicate that swollen and crumpled polymer regions coexist. (b) Similar maps obtained from the same simulations, but showing the standard deviation of the bead separations rather than the mean. Each map is obtained from a single simulation. This gives an indication of how dynamic different polymer regions are, with brighter colours indicating that the separation of the pair of beads varies during the simulation.

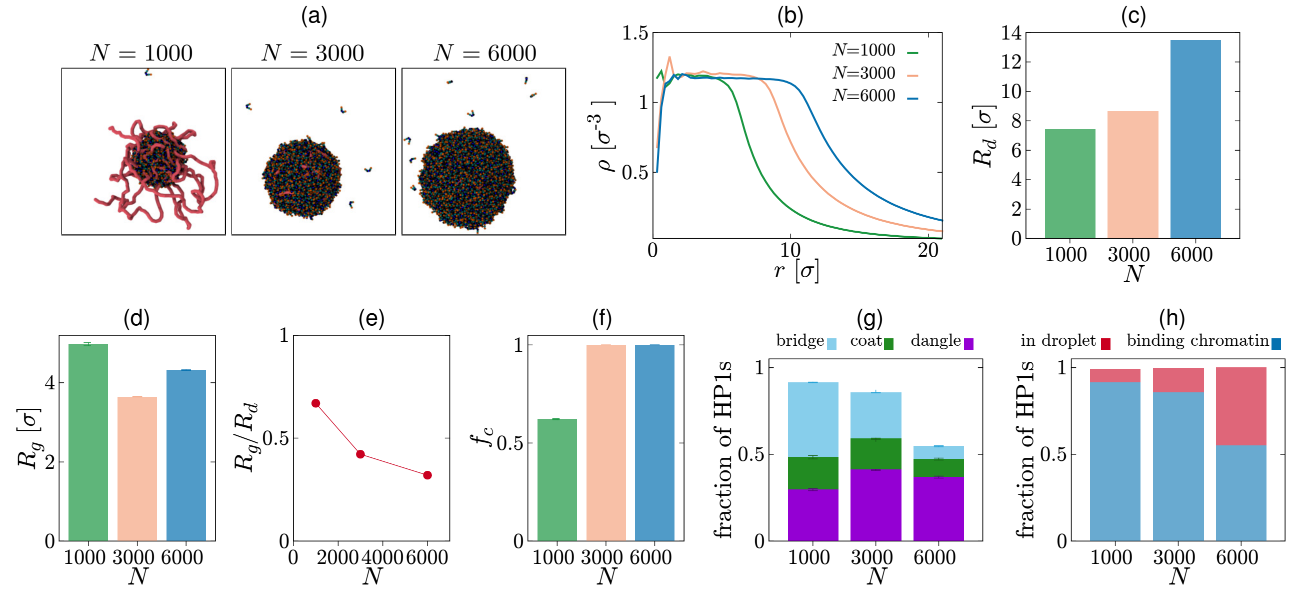

FIGURE S8. Varying the number of proteins for parameters where a droplet would also form without chromatin. Plots showing the effect of varying the number of proteins for parameter values $\epsilon_{\text{HH}} = 6k_BT$, $\epsilon_{\text{HC}} = 14k_BT$ within the absorbing droplet phase. (a) Snapshots from simulations with N = 1000, 3000 and 6000 proteins. (b) Density of proteins within a probing sphere of radius r centred on the the centre of mass of the droplet. (c) Bar plot showing the radius of the protein droplet in simulations with different values of N. (d) Bar plot showing the radius of gyration of the polymer. (e) Plot showing how the ratio R_g/R_d varies with N. (f) Bar plot showing the fraction of polymer beads bound by proteins. (g) Bar plot showing the fraction of the total number of proteins which are bound to the polymer in each of the three modes. Bars are stacked on top of each other so, for example, the distance between the bottom and top of the green region gives the fraction of proteins bound in the coating mode. The total height shows the total fraction of proteins bound to the polymer. (h) Stacked bar plot showing the fraction of the total number of proteins which are in the droplet but not binding to chromatin beads (red) and in the droplet *and* binding to chromatin beads (blue).

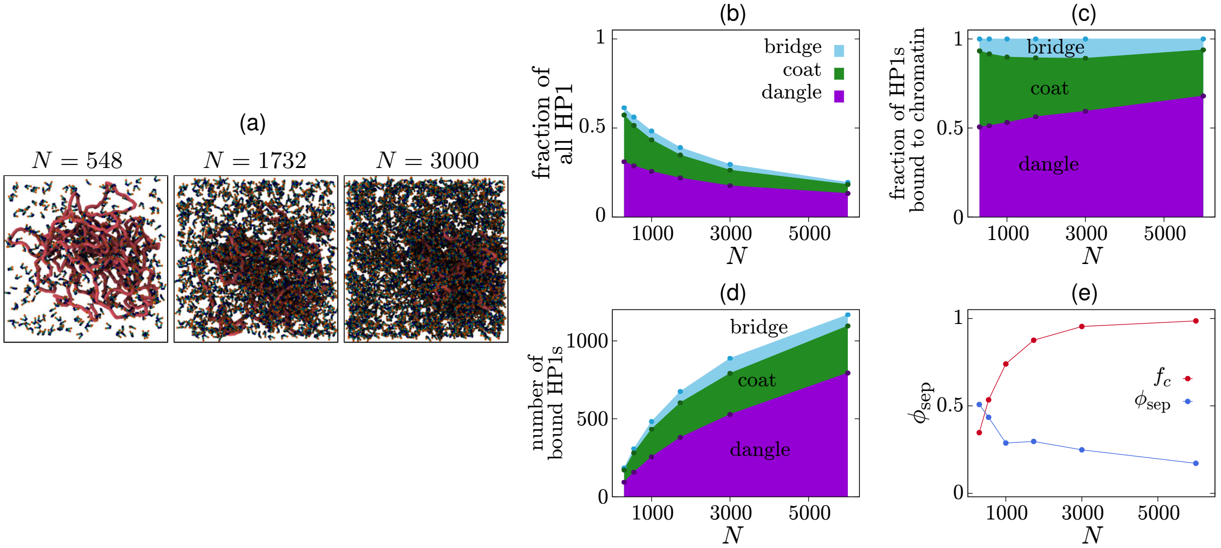

FIGURE S9. Varying the number of proteins for parameters within the coating regime. Plots showing the effect of varying the number of proteins for parameter values $\epsilon_{HH} = 2k_BT$, $\epsilon_{HC} = 18k_BT$, within the coating phase. (a) The fractions of the total number of proteins which are bound to the polymer in the bridging, coating and dangling modes are shown as a function of N. Curves are stacked on top of each other, so for example, the distance between the bottom and top of the green region gives the fraction of proteins bound in the coating mode. (b) A similar plot shows the number of bridging, coating and dangling proteins as a fraction of the number of proteins which are bound to the polymer. (c) Plot showing the total numbers of bridging, coating and dangling proteins. (d) Plot showing the fraction of polymer beads bound by proteins and separation depth parameter as a function of N.

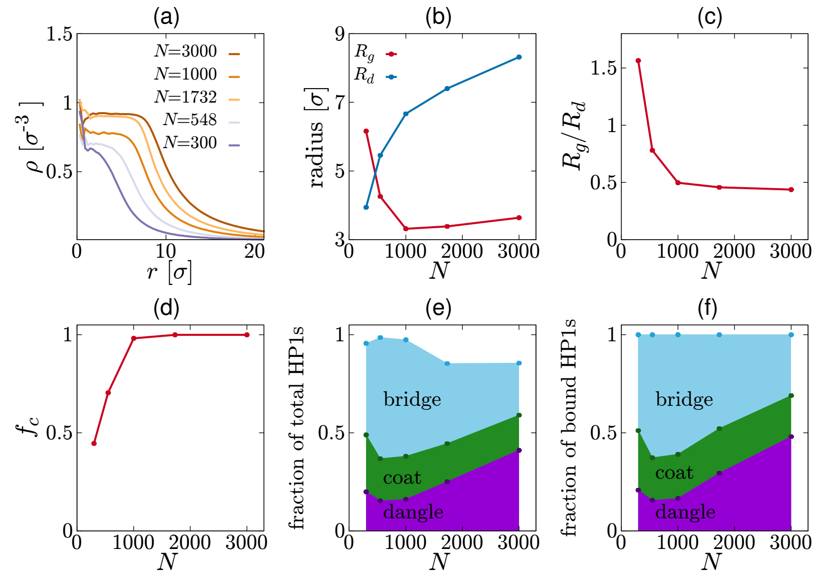

FIGURE S10. Varying the number of proteins for parameters where a droplet *only* forms in the presence of chromatin. Plots showing the effect of varying the number of proteins N for parameter values $\epsilon_{HH} = 4k_BT$, $\epsilon_{HC} = 20k_BT$ within the absorbing droplet phase, in the regime where phase separation would not occur in the absence of chromatin. (a) The density of proteins is measured within a probing sphere of radius r centred on the centre of mass of the protein droplet. (b) Plot showing the radius of the droplet and the radius of gyration of the polymer as a function of N [the same plot is shown with a log-scale in Fig. 4(g) in the main text]. (c) Plot showing how the ratio R_g/R_d varies with N. (d) Plot showing the fraction of polymer beads bound by proteins as a function of N. (e) The fraction of proteins which are bound to the polymer in the bridging, coating and dangling modes are shown as a function of N. Curves are stacked on top of each other, so for example, the distance between the bottom and top of the green region gives the fraction of proteins bound in the coating mode. (f) A similar plot shows bridging, coating and dangling proteins as a fraction of the number of proteins which are bound to the polymer.

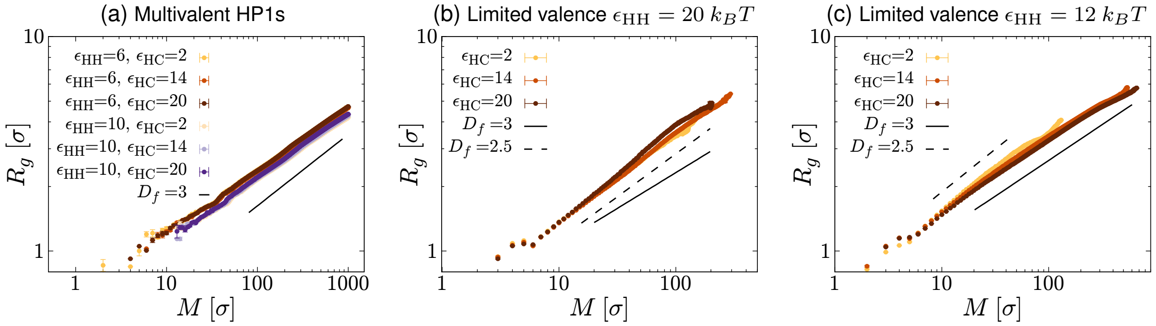

FIGURE S11. Fractal dimension of HP1 sub-clusters Logarithmic scale plots showing radius of gyration R_g *versus* the number of proteins M in sub-clusters for multivalent and limited valence simulations in droplet/cluster regimes [as detailed in Supporting text, section 10]. Each point shows the mean R_g of all unique sub-clusters with a given M; averages are also over time for a single simulation. Error bars show the standard error in the mean (these are often smaller than the points). We expect a power-law relationship with $R_g \sim M^{1/D_f}$, where D_f is the fractal dimension. (a) Points show data obtained from simulations of the multivalent model in the droplet or absorbing droplet regimes, with ϵ_{HH} and ϵ_{HC} as indicated (units are k_BT). Lines show the slope for the indicated values of D_f (b) Points show data obtained from simulations of the limited valence model with large $\epsilon_{HH} = 20k_BT$ and ϵ_{HC} as indicated (clustering regimes). (c) Points show data from simulations of the limited valence model with smaller $\epsilon_{HH} = 12k_BT$.

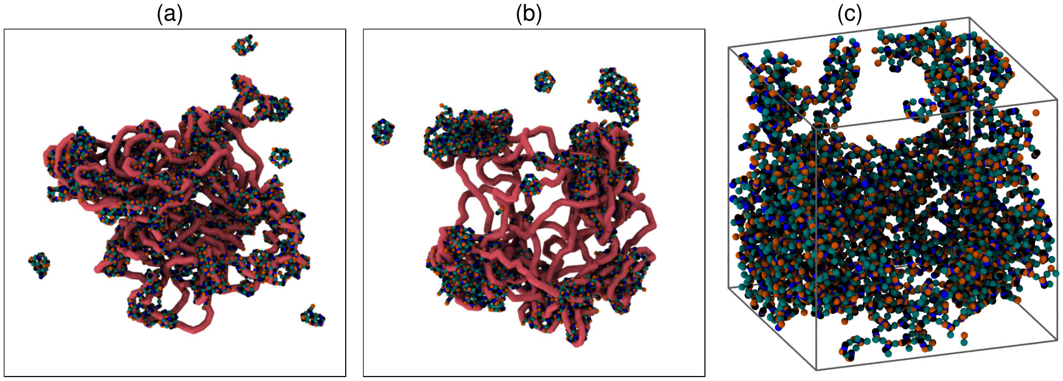

FIGURE S12. Alternative quench schemes lead to different long-lived metastable configurations for limited valence model proteins. Snapshots are shown from simulations of the limited valence model using parameters $\epsilon_{HH} = 16k_BT$ and $\epsilon_{HC} = 16k_BT$. (a) Configuration obtained from the end of a $2 \times 10^4\tau$ long simulation where for the first $10^4\tau$ the HP1-HP1 attraction was switched off; the HP1-chromatin attraction was kept switched on for the full duration. (b) Configuration obtained from the end of a $2 \times 10^4\tau$ simulation where for the first $10^4\tau$ the HP1-chromatin attraction was switched off and instead the HP1-HP1 attraction was on for the full duration. (c) Configuration from a simulation of duration $4 \times 10^3\tau$ with a higher density of HP1s in the absence of polymer ($N = 1000$ proteins in a smaller system of side $l_x = 21\sigma$) using periodic boundary conditions (instead of 'walls' as in all other simulations).

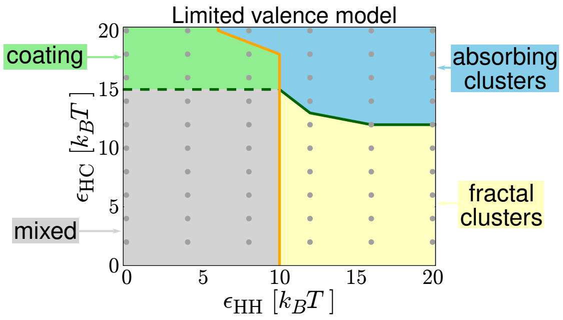

FIGURE S13. Phase diagram for limited valence model HP1s. Phase diagram showing the different behaviours of the limited valence HP1s with different parameter values. Colours correspond to those used in the equivalent regimes for the multivalent case shown in Fig. 2 in the main text. Grey points indicate parameters used in different simulations. The position of the orange line is determined using $\phi_{\rm sep} = 0.5$ as a threshold, while the position of the solid green line is determined by the total fraction of proteins bound to the polymer $f_{\rm tot}$ (we define the absorbing clusters regime as where $\phi_{\rm sep} \geq 0.5$ and $f_{\rm tot} \geq 0.5$). As with the multivalent case, $\phi_{\rm sep}$ is approximately independent of $\epsilon_{\rm HC}$ in the mixed phase, and we set the position of the dashed green line as the point where $\phi_{\rm sep}$ first starts to increase with $\epsilon_{\rm HC}$.

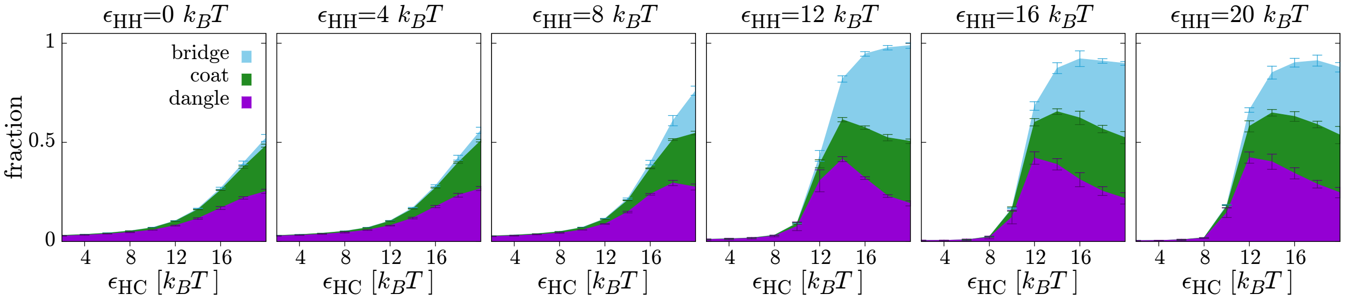

FIGURE S14. Protein-chromatin binding modes for the limited valence protein model. Plots showing the fraction of the $N = 1000$ proteins bound to the chromatin in each mode for different interaction energies. The height of each coloured region indicates the proportion of proteins, with the regions stacked on top of each other. In this way the height of the total coloured region indicates the fraction of proteins bound in any mode $f_{\rm tot}$. Values are obtained from averaging over 4 simulations of duration $10^4\tau$, and error bars show the standard error of the mean.

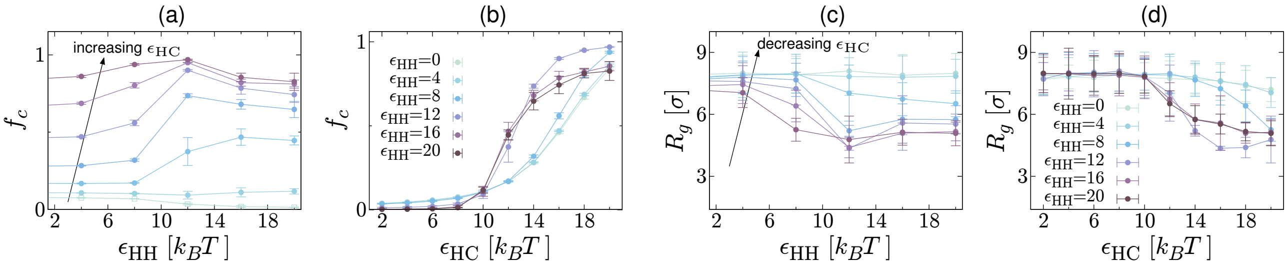

FIGURE S15. HP1-chromatin interactions and compaction for the limited valence protein model. (a-b) Plots showing how the fraction of chromatin beads which are bound by proteins f_c, depends on the interaction energies. In (a) from bottom to top curves are for $\epsilon_{\rm HC}$ between 8 and $20k_BT$ increasing in steps of $2k_BT$. Points are obtained from an average of 4 independent simulations of equilibrium configurations; error bars show the standard error of the mean, and connecting lines are drawn as a guide to the eye. (c-d) Plots showing how the radius of gyration of the polymer representing the chromatin segment depends on the interaction energies. In (c), from top to bottom, curves are again for $\epsilon_{\rm HC}$ between 8 and 20 k_BT increasing in steps of $2k_BT$.

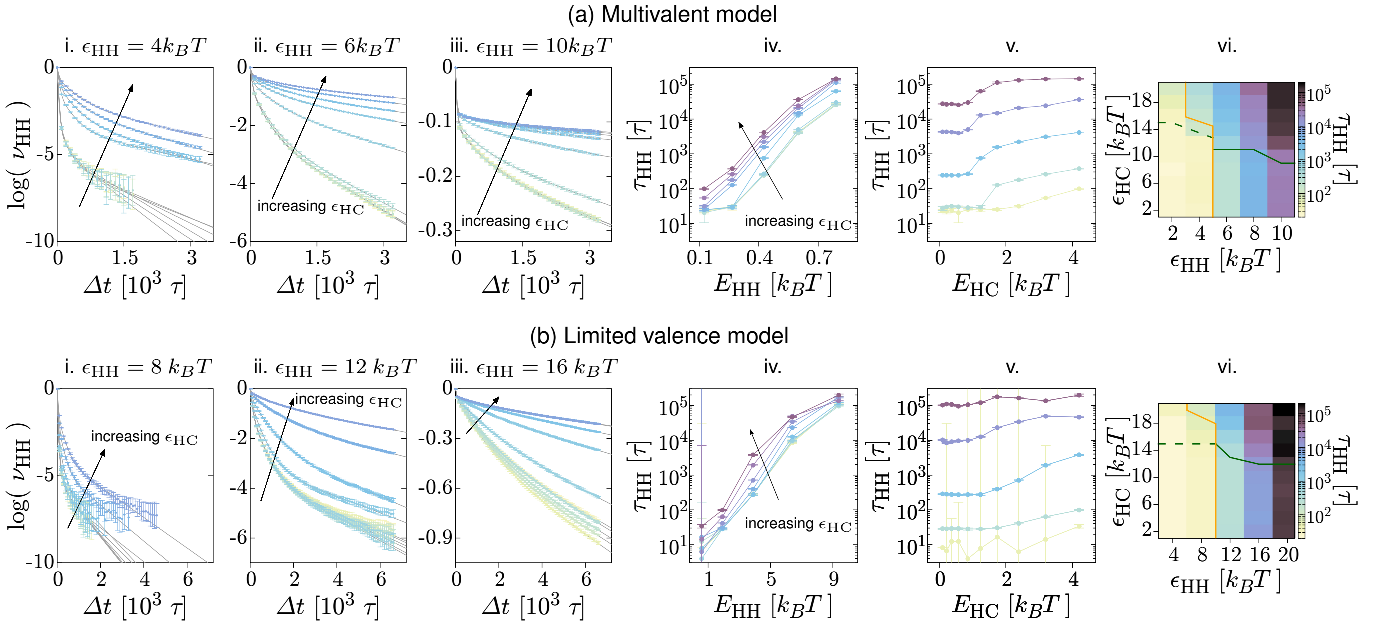

FIGURE S16. Protein dynamics measurements. Results for (a) multivalent model proteins, and (b) limited valence model proteins. i-iii. Plots showing $\nu_{\rm HH}$ as a function of the time interval Δt on a log-linear scale. Coloured points are obtained from 4 independent simulations with error bars showing the standard error. Grey lines show fits to Eq. (9). Where $\nu_{\rm HH}$ gets very small [e.g., in (a)i] the errors can become quite large; we do not show points where the fractional error is larger than 1. iv-v. Plots showing the values of $\tau_{\rm HH}$ as extracted from the $\nu_{\rm HH}(\Delta t)$ fits (see Supporting text, section 12) at different energy parameter values. Here we plot against the effective energies $E_{\rm HH}$ and $E_{\rm HC}$, as defined in Supporting text, section 4. We note that the relationships look very similar to those found for $\epsilon_{\rm HH}$ and $\epsilon_{\rm HC}$ as plotted in Fig. 6 in the main text. Points with error bars are obtained from averages over 4 independent simulations, while lines are added as a guide to the eye. vi. Colour maps showing how $\tau_{\rm HH}$ varies with $\epsilon_{\rm HH}$ and $\epsilon_{\rm HC}$. Lines separating the various regimes are overlaid as in Fig. 2(b) in the main text and Fig. S13.

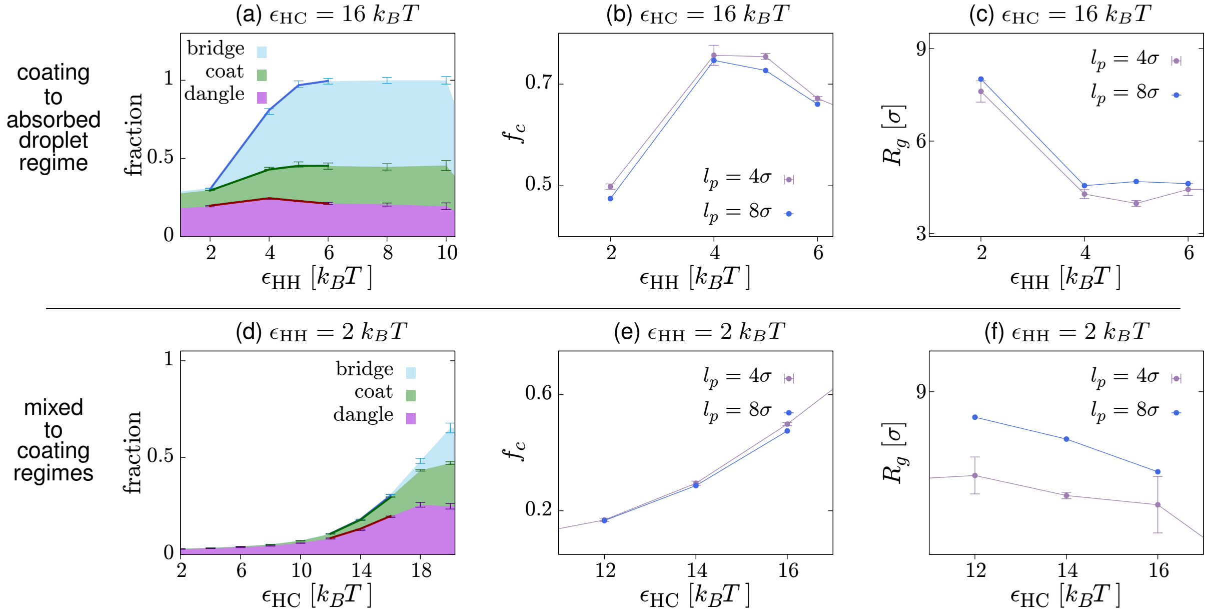

FIGURE S17. Effect of increased polymer stiffness. Panels (a-c) relate to simulations with parameters that span the crossover between the coating and absorbed droplet regimes ($\epsilon_{\rm HC} = 16\ k_BT$). (a) Plot showing the fraction of the $N = 1000$ proteins binding in the bridging, coating and dangling modes. Shaded regions show results for a polymer of persistence length $l_p = 4$, while the solid thick lines show those for simulations with a polymer of persistence length $l_p = 8$ (HP1-HP1 attraction strengths of $\epsilon_{\rm HH} = 2, 4, 5$, and $6\ k_BT$). (b) Plot showing the fraction of polymer beads bound by HP1s as a function of $\epsilon_{\rm HH}$ from simulations with different persistence lengths. (c) Plot showing the polymer radius of gyration as a function of $\epsilon_{\rm HH}$ from simulations with different persistence lengths. Panels (d-f) show similar plots for simulations with parameters that span the crossover between the mixed and coating regimes ($\epsilon_{\rm HH} = 2\ k_BT$).

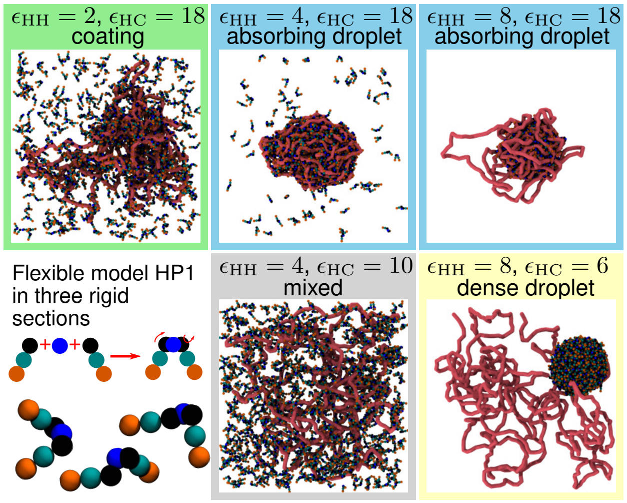

FIGURE S18. Multivalent HP1 dimers with flexible hinges. Bottom left: a schematic shows how a model HP1 dimer is constructed from three rigid components which are connected by springs. These can then rotate freely about each other; snapshots of three possible configurations are shown. Green, blue yellow, and grey framed snapshots are from simulations with the indicated interaction energies (units are k_BT). The same regimes of behaviour are observed as for the fully rigid model HP1 dimers.

Supporting Text

1 CHROMATIN MODEL

In our coarse grained scheme the chromatin fibre is modelled as a chain of L spherical beads of diameter σ, and the position of the ith bead is denoted by $\mathbf{r}_i$. Consecutive beads along the chain are connected by finitely extensible non-linear elastic (FENE) bonds described by

$$U_{\mathrm{FENE}}(r_{i,i+1}) = U_{\mathrm{WCA}}(r_{i,i+1}) + \\ - \frac{K_{\mathrm{FENE}} R_0^2}{2} \log\left[1 - \left(\frac{r_{i,i+1}}{R_0}\right)^2\right], \tag{1}$$

where $r_{i,i+1} = |\mathbf{r}_{i+1} - \mathbf{r}_i|$ is the bead separation, and we set the bond energy $K_{\mathrm{FENE}} = 30k_{\mathrm{B}}T$, and maximum extension $R_0 = 1.6\sigma$. The Boltzmann constant and the temperature are denoted k_B and T respectively, and we use energy units of k_BT throughout. The first term in Eq. (1) is the Weeks-Chandler-Andersen (WCA) potential

$$\frac{U_{\mathrm{WCA}}(r_{ij})}{k_BT} = \begin{cases} 4\left[\left(\frac{d_{ij}}{r_{ij}}\right)^{12} - \left(\frac{d_{ij}}{r_{ij}}\right)^{6}\right] + 1 & r_{ij} < 2^{1/6} d_{ij}, \\ 0 & \text{otherwise,} \end{cases} \tag{2}$$

where d_{ij} is the mean of the diameters of the beads i and j (i.e., $d_{ij} = \sigma$ for two polymer beads). This gives a purely steric interaction preventing beads from overlapping. Non-adjacent beads also interact via the WCA.

To model the bending rigidity of the fibre, the Kratky-Porod potential is introduced between triplets of adjacent beads

$$U_{\mathrm{BEND}}(\theta_i) = K_{\mathrm{BEND}}\left[1 - \cos(\theta_i)\right], \tag{3}$$

where θ_i is the angle formed between beads $i-1$, i and $i+1$, as given by

$$\cos(\theta_i) = \frac{[\mathbf{r}_i - \mathbf{r}_{i-1}] \cdot [\mathbf{r}_{i+1} - \mathbf{r}_i]}{|[\mathbf{r}_i - \mathbf{r}_{i-1}] \cdot [\mathbf{r}_{i+1} - \mathbf{r}_i]|}, \tag{4}$$

and K_{BEND} is the bending energy. The persistence length in units of σ is given by $l_p = K_{\mathrm{BEND}}/k_BT$, and we set $l_p = 4\sigma$. A typical mapping of the polymer model to a chromosome fragment is to consider one bead to represent approximately 10 nm (about 1kbp or 4–5 nucleosomes) of chromatin; then $l_p \simeq 120$ nm, which is reasonable for chromatin (1).

2 HP1 DIMER MODELS

As detailed in the main text, each HP1 dimer is represented by a rigid body consisting of seven spheres arranged as shown in Fig. 1(a): the blue sphere represents the CSD from each HP1 making up the dimer (i.e., two CSDs), then the black, green and orange spheres represent the hinge, CD and NTE domains respectively, two of each per dimer. Each sphere has diameter 0.5σ, with position coordinates relative to the CSD sphere as shown in Fig. S1. The length of the model dimer is 1-1.5σ, equivalent to about 10-15 nm; this is compared to real HP1 dimers which have length 13-22 nm, depending on post-translational modifications (2). The CSD and CD, which are globular domains, are estimated to have a diameter of about 3 nm in reality (3); the sizes of the flexible hinge and NTE domains are more difficult to estimate, but one might expect them to be larger than the folded domains. Choosing to represent the domains as spheres of the same size is therefore an approximation, but we do not expect this will qualitatively change the observed behaviour.

Interactions between HP1 component beads and chromatin beads are modelled as follows. CDs interact attractively with chromatin beads through the potential

$$U_{\mathrm{CD-C}}(r) = \begin{cases} \epsilon_{\mathrm{HC}}\left[(e^{-2\alpha r} - 2e^{-\alpha r}) \\ \qquad -(e^{-2\alpha r_{\mathrm{HC}}} - 2e^{-\alpha r_{\mathrm{HC}}})\right] & r \le r_{\mathrm{HC}}, \\ 0 & \text{otherwise,} \end{cases} \tag{5}$$

where r is the separation between the centres of the CD and the chromatin bead, ϵ_{HC} is the energy which determines the strength of the interaction, α is a "shape" parameter, and r_{HC} is the cut-off distance which sets the range of the interaction. We set $\alpha = 5$ and $r_{\mathrm{HC}} = 0.9\sigma$. The CSD, hinge, and NTE interact sterically with the chromatin beads, through the WCA potential given in (2); we set $d_{ij} = 0.75\sigma$ for the CSD and $d_{ij} = 0.5\sigma$ for the hinge and NTE. This allows partial overlap of these beads with the chromatin beads, which is essential to permit interaction between the CDs and chromatin beads (it also accounts for the hinge and NTE being flexible/disordered domains).

Interactions between HP1s are modelled similarly. The hinge and NTE domains in different HP1 dimers interact via the potential

$$U_{\mathrm{h-NTE}}(r) = \begin{cases} \epsilon_{\mathrm{HH}}\left[(e^{-2\alpha r} - 2e^{-\alpha r}) \\ \qquad -(e^{-2\alpha r_{\mathrm{HH}}} - 2e^{-\alpha r_{\mathrm{HH}}})\right] & r \le r_{\mathrm{HH}}, \\ 0 & \text{otherwise.} \end{cases} \tag{6}$$

The two different models of the HP1-HP1 interaction are specified by different sets of parameters: for the *multivalent* model we set $\alpha = 0.5$ and $r_{\mathrm{HH}} = 1.3\sigma$; for the *limited valence* model we set $\alpha = 5$ and $r_{\mathrm{HH}} = 0.6\sigma$. All other HP1 component beads interact sterically via the WCA, except for the CSD and the NTE which can overlap (there is no interaction) in order to permit the correct binding of NTE domains with hinges.

We note that the minima of the functions $U_{\mathrm{CD-C}}(r)$ and $U_{\mathrm{h-NTE}}(r)$ is at $r = 0$, i.e., the beads can overlap. In practice, it is not always possible to achieve the separation $r = 0$ due to steric interactions between the other component beads. For the HP1-chromatin interaction, allowing the CD and chromatin beads to overlap, along with the short range of the interaction, ensures that a given CD can interact with at most one chromatin bead at a time (e.g., a single CD cannot form a bridge between two chromatin beads). Since the CD spheres are smaller than the polymer beads, it is possible for more than one CD to interact with a given polymer bead at the same time. These are reasonable choices, since a chromatin bead represents several nucleosomes, and the CD is thought to interact with nucleosome surface charges. We also note that the functional forms of $U_{\mathrm{CD-C}}(r)$ and $U_{\mathrm{h-NTE}}(r)$ are the same as the commonly used Morse potential.

In the *limited valence* case, the short range of $U_{\mathrm{h-NTE}}(r)$ with no repulsive core means that one NTE can interact with exactly one

CSE at a time and *vice versa*. For the *multivalent* case the longer range and small value of α (which leads to a broader shape) is such that multiple NTEs can simultaneously interact with one CSE and *vice versa*.

3 LANGEVIN DYNAMICS

We use the LAMMPS software (4) to perform Langevin dynamics simulations. Briefly, the position of each polymer bead and the centre of mass of each HP1 dimer is governed by the equation

$$m_i \frac{d^2\mathbf{r}_i}{dt^2} = -\nabla U_i - \xi_i \frac{d\mathbf{r}_i}{dt} + \sqrt{2k_BT\xi_i}\boldsymbol{\eta}_i(t), \quad (7)$$

where $\mathbf{r}_i$ is the position of the centre of mass of the ith bead or HP1, m_i is its mass, U_i is the sum of all the interaction potentials for object i. The friction ξ_i sets the diffusion constant $D_i = k_BT/\xi_i$ for each object; we set $m_i = 1$ and $\xi_i = 0.5$ for polymer beads; for simplicity, we also set the mass of HP1 component beads to 1, and the friction for the HP1 rigid bodies to 0.5. The vector $\boldsymbol{\eta}_i(t)$ is a noise term with components which satisfy

$$\langle \eta_{i\alpha}(t) \rangle = 0 \;\text{ and }\; \langle \eta_{i\alpha}(t)\eta_{j\beta}(t') \rangle = \delta_{ij}\delta_{\alpha\beta}\delta(t-t'), \quad (8)$$

where $\eta_{i\alpha}$ is component α of the noise vector for object i, and δ_{ij} and $\delta(t)$ are the Kronecker and Dirac delta functions respectively. The orientation of the HP1 rigid bodies is governed by a similar Langevin equation for rotation. These equations are solved using a velocity-Verlet algorithm with time step dt=0.001τ, where τ is the simulation time unit defined by $\tau = \sqrt{m\sigma^2/k_BT}$.
One can make a rough mapping between simulation and real time units by considering the typical time for a polymer bead to diffuse across its own diameter $\tau_{\mathrm{Br}} = \sigma^2/D$. Then using the Stokes-Einstein relation for a sphere $D = k_BT/(3\pi\eta\sigma)$ and taking the diameter to be $\sigma = 10$ nm and the viscosity of nucleoplasm as 10 cP we obtain $\tau \approx 0.456$ ms.

4 INTERACTION ENERGY SCALES

In the previous section we introduced ϵ_{HC} and ϵ_{HH} as the interaction energies for protein-protein and protein-chromatin interactions respectively, and we use these throughout the main text. We note that due to the form of Eq. (6), the energy at the minima of this function is not equal to ϵ_{HC} (the second term shifts the function upwards). The same is true of Eq. (5) and ϵ_{HH}. We further note that while for both functions the minima is at $r = 0$, due to the geometry of the protein (and stetic interactions between the different component beads) the separation may not in practice be able to reach zero. We therefore expect the interactions in our model to have some effective strengths $E_{\mathrm{HC}} \neq \epsilon_{\mathrm{HC}}$ and $E_{\mathrm{HH}} \neq \epsilon_{\mathrm{HH}}$. It is not guaranteed that the relationship between the effective and "bare" interaction energies are linearly related. We therefore measured this empirically from a set of calibration simulations.
A simple assumption is that (in the absence of cooperative interactions) binding events behave as in the Kramer's escape problem (5); in Kramer's approximation, the mean duration of binding is $\langle \tau_{\mathrm{bind}} \rangle = \tau_0 e^{\epsilon/k_BT}$, where ϵ is the interaction energy. τ_0 is the typical time for which the two diffusing objects would remain in contact in the absence of an attraction ("in contact" meaning having separation less than the interaction range). We performed a set of calibration simulations of two HP1 dimers in which one hinge in one dimer was allowed to interact with one NTE in the other. We measured the duration of HP1-HP1 interaction events; Fig. S2(a) left panel shows a plot of the unbinding rate $k_{\mathrm{off}} = \langle \tau_{\mathrm{bind}}^{-1} \rangle$ as a function of ϵ_{HH} for the multivalent HP1 model. We then fit to obtain the function $E_{\mathrm{HH}} = f(\epsilon_{\mathrm{HH}})$, which gives a mapping between the bare and effective interaction energies (Fig. S2(a) right panel). We considered a number of functional forms for $f(\epsilon)$, finding a good fit for $f(\epsilon) = C_1\epsilon - C_2(1 - e^{-\epsilon/C_3})$; best fit values for the constants C_1, C_2 and C_3 are given in the figure. A similar scheme was used in Ref. (6). We repeated the same process for the limited valence HP1 model [Fig. S2(b)].
To calibrate the protein-polymer interactions we performed a similar set of simulations, but with a short 100 bead polymer and a single HP1 dimer (in which only one CD was allowed to bind to the polymer). We measured the unbinding rate k_{off} as a function of E_{HC} [Fig. S2(c) left], and then fit to obtain a function $\epsilon_{\mathrm{HC}} = f(E_{\mathrm{HC}})$. Here the function $f(E) = c_1E + c_2E^{C_3}$ gives a good fit, leading to the energy mapping in Fig. S2(c) right.
In all of the calibration simulations we used a small periodic system size to minimise the time interval between binding events. We used a very long run time (at least $5 \times 10^6\tau$), and we measured the bead separations at every time step to ensure we properly captured the entire binding event time.
As noted in the main text, these measurements are comparable to *in vitro* measurements of HP1 residence times on immobilised H3K9me3 chromatin; experimental values lie in the range 190-250 ms, and depend on the presence of other chromatin modifications (7). In our simulations, the range of ϵ_{HC} values studied gives HP1-chromatin interaction durations between zero and 50 ms; so this is of the right order of magnitude, albeit smaller. The range of ϵ_{HH} values studied gives protein-protein interaction durations between 0 and 1 ms for the case of the multivalent HP1 model. For the limit valence model this was between zero and 14 s.

5 OBTAINING EQUILIBRIUM CONFIGURATIONS

In this work, for the case of the multivalent HP1 proteins we have focused on the equilibrium properties of the system. A common consideration in molecular dynamics simulations is ensuring that trajectories are representative of the equilibrium state. In order to obtain equilibrium configurations we used a specific annealing process, and also performed a number of additional test simulations.
Initially, we started with a configuration in which the polymer followed the path of a random walk, and proteins were positioned at random within the confinement volume. An initial short simulation using purely repulsive "soft" interactions was used to remove bead overlaps; then a longer run was performed using the force field detailed above, but without attractive interactions ($\epsilon_{\mathrm{HC}}, \epsilon_{\mathrm{HH}} = 0$). One scheme is then to instantaneously switch on attractive interactions with the desired energy, i.e., performing an instantaneous quench from high to low temperature. Using this scheme, for large ϵ_{HH} we found that multiple clusters of proteins formed quickly (indicating spinodal decomposition). These clusters proceeded to coarsen via coalescence and Ostwald ripening

(while single proteins can escape from a cluster, we did not observe large clusters breaking into smaller ones). Without any mechanism to arrest cluster coarsening we expect to observe a single cluster at equilibrium; however, these dynamics can be very slow (especially diffusion of large protein clusters), and in general a single cluster could not be obtained within a reasonable simulation time. For intermediate values of $\epsilon_{\rm HH}$, after a quench we often observed a single or small number of clusters form, as would be expected from a nucleation and growth process. Obtaining a single cluster was therefore much quicker in this case.

For the majority of our simulations we therefore employed a different scheme designed to obtain equilibrium (single cluster) configurations more quickly. Specifically, we first switched on the protein-polymer interaction (an instantaneous quench from zero to the desired value); then we slowly increased the protein-protein interaction strength over an extended time. The idea being that the system would first move through the parameter regime where the dynamics follow nucleation and growth (and interaction with the polymer would promote nucleation). We then ran the simulation for long enough to obtain a single cluster. In all cases, once a single cluster configuration was achieved we then used that as an initial configuration for another simulation of length $5 \times 10^3\tau$, from which we obtained our results. All of the results presented for the multivalent HP1 model are obtained from an average over at least 4 independent simulations. We also checked that measured quantities (such as R_g and the number of proteins bound to the polymer in the different modes) were fluctuating about steady values, and were not systematically changing during the simulation.

Although the above scheme generates single-cluster configurations, it is still possible for the system to become stuck in a long-lived metastable configuration not representative of equilibrium, particularly if the interaction energies are large. For example, within the absorbing droplet phase—where the fraction of polymer absorbed, f_c, depends on the interaction energies—it is important to verify that this really is reflective of equilibrium. We performed some additional quench simulations within the absorbing droplet phase, confirming that after a sudden change of parameters f_c relaxes to what we expect is the equilibrium value. In Fig. S3(a) we show on a phase diagram the various quench simulations performed; Figs. S3(b-d) show f_c as a function of time after a quench at $t = 0$. After the quenches at constant values of $\epsilon_{\rm HC}$ [Figs. S3(b,c)], we find that f_c relaxes to the expected value within about $5 \times 10^4\tau$. This verifies that our configurations are representative of equilibrium (measurements of the polymer R_g show similar behaviour). At constant $\epsilon_{\rm HH} = 8k_BT$, after a sudden change in $\epsilon_{\rm HC}$ the relaxation towards the expected value is much slower [Fig. S3(d)]; however, the continuous decrease of f_c suggests that the equilibrium value would be reached in a longer simulation.

Another important parameter regime is at intermediate $\epsilon_{\rm HH} \approx 4\ k_BT$, where the droplet only forms if the protein-chromatin interaction is strong enough. To verify that the droplet is indeed unstable for small $\epsilon_{\rm HC}$, we performed a simulation where the initial condition was an equilibrium configuration obtained at $\epsilon_{\rm HH} = 6k_BT$, $\epsilon_{\rm HC} = 4k_BT$; we then instantaneously reduced the protein-protein interaction to $\epsilon_{\rm HH} = 4\ k_BT$ [purple arrow in Fig. S3(a)]. We observed that the protein cluster breaks apart; this rules out the possibility that droplets are stable for these parameters, but just take a very long time to form.

Finally, for two sets of parameter values within the absorbed droplet phase [green stars in Fig. S3(a)] we sample equilibrium configurations using the replica exchange method (8). During a replica exchange simulation (also known as parallel tempering) a set of N independent simulations are performed in parallel, each at a slightly higher temperature than the last, $T_1 < T_2 < \ldots < T_N$. At regular time intervals, the configurations in simulations i and $i + 1$ are exchanged according to a Monte Carlo update rule. The aim is to more easily sample configurations which are rare at lower temperatures, through the exchange of configurations with replicas at higher temperatures; effectively, this allows the system to escape from local free energy minima. Replica exchange is implemented natively in LAMMPS (4). For each parameter pair we performed a set of 36 simulations at temperatures between 1.0 and 2.05 (units of $m\sigma^2/k_B\tau^2$). We used the end point of our standard simulations as an initial condition, and ran the replica exchange for $10^4\tau$ (exchanges were attempted every 0.1τ, and 70% of attempts were successful). The temperature in each replica simulation was then reduced gradually back to 1.0 over a further 500τ (without further exchanges), before a standard (constant T) simulation of length $5 \times 10^3\tau$ was performed. For both parameter sets, the values of f_c, R_g and $f_{\rm tot}$ were consistent across these simulations and with our original simulations. This suggests that our original shorter simulations are indeed representative of equilibrium, and not a long-lived metastable state.

As noted in the main text, the limited valence HP1s behave more like classic patchy particles which are known to exhibit long-lived dynamically arrested non-equilibrium phases including gels and closed loops (which can form at equilibrium at zero temperature) (9–12). This means that it is more difficult to obtain true equilibrium configurations than in the multivalent case. For the limited valence model, we therefore did not seek to explicitly obtain equilibrium configurations. Instead we ran each simulation for $2 \times 10^4\tau$ after starting from a configuration obtained for $\epsilon_{\rm HH}, \epsilon_{\rm HC} = 0$, taking measurements from the final $10^4\tau$. We then checked that quantities such as f_c, $f_{\rm tot}$ and R_g were not systematically changing during this time. Thus, our simulated structures represent a metastable or dynamically arrested state obtained via a rapid quench from low to high interaction energies.

6 THE $\rho - \epsilon_{\rm HH}$ PHASE DIAGRAM

We return now to the multivalent HP1 model. In order to calculate the protein densities, e.g., within or outside of a protein droplet, we consider a ‘probe sphere’ of radius r centred on the centre of mass of the largest droplet (protein cluster). We then progressively increase r, and calculate the density of proteins within the probe sphere, and within a spherical shell of width dr. We then average over time and repeat simulations (finding a new droplet centre of mass each time).

In Fig. S4 we plot several quantities as a function of the probe sphere radius for representative values of $\epsilon_{\rm HH}$ and $\epsilon_{\rm HC}$. In Fig. S4(a-c) we consider values within the droplet and absorbing droplet regimes, with panel (a) showing the number of proteins within the

probe sphere $N_{\rm ps}$ as a function of radius. In both cases we observe $N_{\rm ps} \sim r^3$ for r smaller than the droplet radius, as expected for a spherical droplet with a uniform density. Fig. S4(b) shows the *local* protein density, calculated as

$$\rho_{\rm shell}(r) = \frac{N_{\rm shell}(r)}{4\pi r^2 dr}$$

where $N_{\rm shell}(r)$ is the number of proteins within the spherical shell of width $dr = 0.3\sigma$ and radius r. We find that although $\rho_{\rm shell}(r)$ is rather noisy, it shows a clear drop to zero when r reaches the droplet radius. In Fig. S4(c) we plot the overall density within the probe sphere $\rho_{\rm ps} = N_{\rm ps}/V_{\rm ps}$ as a function of r, where $N_{\rm ps}(r) = \int_0^r N_{\rm shell}(r')dr'$ and $V_{\rm ps} = (4/3)\pi r^3$. We find that $\rho_{\rm ps}(r)$ is initially approximately constant with r, but at larger values it decreases towards the overall protein density ($\rho = 0.0233\ \sigma^{-3}$). We use the plot in Fig. S4(b) to obtain a lower bound for the droplet radius $r_{\rm in}$ (dashed line); then to obtain an estimate of the density in the protein rich phase, $\rho_{\rm HD}$, we fit a horizontal line to the plot in Fig. S4(c) in the range $\sigma < r < r_{\rm in}$. Similarly, from Fig. S4(b) we can also identify an outer radius, $r_{\rm out}$ (dotted line) which encompasses the entire droplet, plus any interface region or deviation due to the droplet not being exactly spherical. The density in the protein poor phase can then be estimated as $\rho_{\rm LD} = (N - N_{\rm ps}(r_{\rm out}))/(V_{\rm box} - V_{\rm ps})$, where $V_{\rm box}$ is the volume of the simulation box and $N = 1000$ is the total number of proteins. We found that (except in the mixed regime) the choice $r_{\rm in} = 4\sigma$, $r_{\rm out} = 9\sigma$ was appropriate for all energy parameters ($\rho_{\rm ps}$ is approximately constant for $r \le r_{\rm in}$ and scales as r^{-3} for $r \ge r_{\rm out}$); we therefore used these values throughout.

Figures S4(d-f) show similar plots as detailed above, but comparing parameter values from the mixed or absorbing droplet regime. We note that the probing sphere procedure does not make sense in the mixed phase, where clusters of between 4 and 5 HP1s do form but only transiently: a different transient 'largest cluster' is identified at each time point. We therefore do not consider the mixed regime results further. The $\epsilon_{\rm HH} = 4k_BT$, $\epsilon_{\rm HC} = 16k_BT$ case is in the absorbing droplet regime, and we note that the $\rho_{\rm ps}(r)$ curve in Fig. S4(e) shows a slower drop off with r at the droplet radius than that in Fig. S4(b). The reason for this is that for an absorbing droplet, a significant fraction of the polymer extends out from the drop, and can be bound by coating proteins; this leads to a broader interface region. Figures S4(g-i) show plots comparing the mixed and coating regimes. In the latter case the $\rho_{\rm ps}(r)$ curve is even broader; nevertheless, we can still estimate $\rho_{\rm HD}$ and $\rho_{\rm LD}$ using the above procedure.

For a given value of $\epsilon_{\rm HC}$, we can plot values of $\rho_{\rm HD}$ and $\rho_{\rm LD}$ on the ρ-$k_BT/\epsilon_{\rm HH}$ plane to obtain the more conventional phase diagram used for phase separating systems. Such plots are shown in Figs. S5(a-b). By only including points for parameters where $\phi_{\rm sep} > 0.6$ we can identify parts of the boundary between droplet and non-droplet phases [Fig. S5(a)]. By reducing this threshold to $\phi_{\rm sep} > 0.2$ [Fig. S5(b)], we can also estimate boundaries for the coating regime, where we observe a partial phase separation (local increase in protein density due to coating the polymer as discussed in the main text). This allows us to sketch out the phase diagram for small and large values of $\epsilon_{\rm HC}$ in Fig. 1(c) in the main text, reproduced in Fig. S5(c) for completeness. As detailed in the main text and in Supporting text, section 9 below, there is also a region [shaded bar in Fig. S5(c)] where a droplet only forms due to the presence of the polymer. Here the density of proteins within the two phases varies with the protein concentration.

7 HYSTERESIS IN THE 'DROPLET'–'ABSORBING DROPLET' TRANSITION

In Figs. 3 and 4 of the main text we showed that there is an abrupt change in quantities such as the total fraction of proteins bound to the polymer $f_{\rm tot}$, the fraction of polymer beads bound by proteins f_c, and the polymer radius of gyration R_g, as $\epsilon_{\rm HC}$ is increased and the system moves from the droplet to the absorbing droplet regime. This hints that there may be a first order phase transition in the thermodynamic limit. To elucidate this further, we performed simulations where we slowly vary the parameter values in time and looked for evidence of hysteresis. In Fig. 3(c) in the main text we show a hysteresis loop for the fraction of proteins bound to the polymer (in total and in different modes) as $\epsilon_{\rm HC}$ is slowly increased from $8k_BT$ to $14k_BT$ before being decreased again.

To obtain this we ran 12 independent repeat simulations for $4\times10^4\tau$, each starting from a different equilibrium configuration for $\epsilon_{\rm HH} = 6k_BT$ and $\epsilon_{\rm HC} = 8k_BT$ (droplet regime). For the first $2\times10^4\tau$ of each simulation $\epsilon_{\rm HC}$ is increased by an increment of $3\times10^{-2}k_BT$ every $10^2\tau$, until it reaches $\epsilon_{\rm HC} = 14k_BT$ (the absorbing droplet regime). Then, over the second $2\times10^4\tau$ of the simulation $\epsilon_{\rm HC}$ is reduced in the same fashion (until $\epsilon_{\rm HC} = 8k_BT$). We keep the protein-protein interaction energy constant throughout at $\epsilon_{\rm HH} = 6k_BT$. For this intermediate value the droplet is highly dynamic (proteins often change their neighbours and there is relatively fast exchange of proteins between the droplet and the surrounding low density region, see Fig. 6 in the main text); one would expect a slower response for larger $\epsilon_{\rm HH}$.

In Fig. S6 we show how R_g varies during the same simulations alongside the hysteresis plots for the fraction of proteins binding in different modes. This shows that there is also hysteresis in terms of the polymer configuration, as it retains memory of its previous state for a significantly long time after the system crosses the transition.

8 POLYMER DISTANCE MAPS

To examine the polymer structure we can plot a 'distance map' showing the mean 3D distance between every pair of polymer beads. Figure S7(a) shows maps from multivalent HP1 simulations representative of different regions of the phase diagram [Fig. 2(c) in the main text]. Each map is obtained from a single simulation of duration $5\times10^3\tau$. In the mixed and dense droplet regimes (not shown), distances between chromatin beads which are separated along the chain tend to be large and the configuration is dynamic. In the coating regime [Fig. S7(a)i], the chromatin is swollen, thus relative distances are again large, even though some distant regions along the chromatin chain can be connected by the very small fraction of HP1s which bind in the bridging mode. On the distance map this can be seen as a mixture of bright (large distance) and dark (short distance) regions.

In the absorbing droplet regime the distance map differs for

different interaction energies. When ϵ_{HC} is large but ϵ_{HH} has an intermediate value [Fig. S7(a)ii], all of the chromatin is absorbed into the droplet in a crumpled configuration. Distances between chromatin beads which are separated along the chain tend to be short (dark colours in the map). If both interaction energies are large [Fig. S7(a)iii], the map shows mainly short distances but with a few bright stripes; the latter are the short regions of the chromatin which extend out of the droplet, and so tend to be further away from the rest of the polymer. Figure S7(a)iv shows the case of large ϵ_{HH} but intermediate ϵ_{HC}. Here, quite long polymer segments loop out from the droplet: the map shows a coexistence of brighter regions (swollen chromatin) and darker regions (crumpled chromatin).

Figure S7(b) shows similar maps, but the standard deviation of the distance between beads is plotted instead of the mean. This gives a measure of how dynamic the polymer configuration is, with a larger standard deviation indicating more variation of the polymer bead separation in time (recall that each map is obtained from a single simulation run). Figures S7(b)iii-iv show that some regions have a strikingly low standard deviation (black); these corresponding to regions absorbed within the protein droplet. This implies little variation in polymer bead separations within the droplet (slow dynamics), with large variation of separation in the protruding loops (faster dynamics). For the parameters used in Fig. S7(b)ii, a fully absorbing (no protruding loops) droplet forms; here the variation in distances is more uniform and in the intermediate range, implying that the polymer is mobile within the droplet.

9 VARYING PROTEIN DENSITY

As detailed in the main text, we performed simulations with different numbers of multivalent HP1s at three pairs of ϵ_{HH},ϵ_{HC} parameter values within the absorbing droplet regime.

Figure S8 shows results for the case where $\epsilon_{HH} = 6k_BT$ and $\epsilon_{HC} = 14k_BT$; for these parameters the protein droplet would form even in the absence of chromatin. As can be observed from the snapshots in Fig. S8(a), increasing the number of proteins leads to a larger droplet which absorbs a larger fraction of the polymer. We confirm quantitatively that the density of HP1s within the droplet is independent of the total number of HP1s (i.e., the overall density) using the probing sphere procedure detailed in Supporting text, section 6 above. We plot the density within the probe sphere ρ_{ps} as a function of its radius r in Fig. S8(b), while the droplet radius for the three different values of N is shown in Fig. S8(c) [taken to be the position of the half maximum point in the $\rho_{ps}(r)$ curve]. From this we see that when HP1-HP1 attraction drives droplet formation the behaviour is consistent with standard (Model-B) phase separation (ρ_{HD} is independent of N and droplet radius R_d increases as $N^{1/3}$).

Figures S8(d-h) reveal a complicated relationship between the droplet size and chromatin absorption/compaction. This stems from the balance between the energetic gain which arises from HP1s binding chromatin, the entropic loss due to HP1 bound chromatin being confined to the volume of the droplet, and any energetic loss due to HP1-HP1 'bonds' being broken to accommodate HP1-chromatin 'bonds'. For the $N = 6000$ case the polymer is completely absorbed within the droplet, and we note that the volume which the polymer coil occupies is significantly smaller than the volume of the droplet. That is to say, the polymer is compacted to a greater extent that it would be due to simply being confined within the droplet. This is clear if one considers the ratio R_g/R_d, which steadily decreases with N [Fig. S8(e)]. To understand this, we varied the interaction strengths ϵ_{HH} and ϵ_{HC} by a small amount (such that we stay in the same regime) and observed the effect on the polymer radius of gyration (data not shown). We found that increasing ϵ_{HH} led to greater compaction of the polymer. This is consistent with expectations if we consider the protein droplet to be an effective solvent within which the polymer is dissolved (e.g., as considered in Flory-Huggins theory). On the other hand, increasing ϵ_{HC} also led to greater compaction of the polymer; in the Flory-Huggins theory, increasing polymer-solvent attraction leads to *swelling* of a polymer. Clearly the ability of our model HP1s to form bridges means that treating the droplet as a solvent gives an incomplete picture.

In Figure S9 we present results for the $\epsilon_{HH} = 2k_BT$, $\epsilon_{HC} = 18k_BT$ case, which is within the coating regime. Here, changing the number of proteins does not qualitatively change the behaviour. As expected, as N increases, the number of proteins bound to the chromatin increases [initially steeply, but this levels off as the polymer become saturated, Fig. S10(c-d)]. The majority of proteins bind in the coating and dangling modes [Fig. S10(a-b)]; the bridging binding mode is always disfavoured, even for large N. A plot showing the *total* number of bridging, coating and dangling HP1s [Fig. S10(c)] reveals that there is a slight increase of the total number of bridges as N increases, but there is still negligible reduction in polymer radius of gyration compared to the mixed regime. The degree of phase separation remains low (small but non-zero ϕ_{sep}), though ϕ_{sep} initially *decreases* with N, as the way the additional proteins distribute between the low and high density regions is complex.

Figure S10 shows some additional results for the $\epsilon_{HH} = 4k_BT$, $\epsilon_{HC} = 20k_BT$ case (as in Fig. 4 in the main text). This is the absorbing droplet regime, but here the droplet can only form in the presence of chromatin. In Fig. S10(a) we again consider a probing sphere of radius r and plot $\rho_{ps}(r)$, from which the droplet density ρ_{HD} is obtained (following the scheme described in Supporting text, section 6). Figure S10(b) shows how the droplet radius and polymer radius of gyration varies with N, as in Fig. 4(g) in the main text, but here on a linear rather than logarithmic scale. As above, we find that the ratio R_g/R_d decreases as N increases [Fig. S10(d)], but reaches a plateau as the growth of the droplet radius with N slows.

10 DROPLET FRACTAL DIMENSION IN THE MULTIVALENT AND LIMITED VALENCE MODELS.

In the snapshots of the limited valance model shown in Fig. 5 in the main text, we observe irregularly shaped protein clusters with a structure strikingly different to the spherical droplets formed by the multivalent model. To quantify this difference, here we estimate the fractal dimension D_f of the clusters. In simulations,

the fractal dimension of a cluster, e.g. from a diffusion limited cluster aggregation (DLCA) process (13), is typically obtained from a scatter plot of the cluster mass (or number or particles) *versus* radius. For spherical clusters one would expect a scaling $R \sim N^{1/3}$, while fractal clusters give $R \sim N^{1/D_f}$ where $D_f < 3$ for a 3D system.

In our simulations, we typically observe a single or small number of clusters, meaning it is difficult to obtain enough measurements to determine D_f. Another common method is to extract the fractal dimension from the structure factor $S(q)$ (14), but this is again difficult to obtain from our simulations of a small number of clusters in a confined geometry. To estimate D_f, we instead consider smaller regions of the clusters, or "sub-clusters", measuring their mass and radius of gyration. We use the following scheme: we consider the ith HP1 together with all of its bound neighbours (defined as any HP1 whose centre of mass is within 1.1σ of HP1 i; different threshold values do not significantly alter the result). We denote this set of proteins a level 1 sub-cluster, and record the number of proteins M and radius of gyration R_g associated with this set. This is repeated for all HP1s in the system. We then consider level 2 sub-clusters, consisting of HP1 i, its bound neighbours, and all of the bound neighbours of neighbours; again, we record M and R_g for $i = 1 \dots N$. Level 3 sub-clusters include neighbours of neighbours of neighbours, etc. We continue increasing the level until there are no further unique sub-clusters, taking care not to double count. Figure S11 shows plots of R_g against M for all possible sub-clusters; each point represents the mean R_g of all sub-clusters (of any level) with a given number of HP1s, M.

For the multivalent model, for all parameters where there are protein droplets, the sub-cluster plots are roughly linear on a log-log scale, and have similar slope. A linear fit to a function $R_g = aM^{1/D_f}$ gives $D_f \approx 3.2$, close to the expected $D_f = 3$ for spherical droplets.

For the limited valence model, we find that sub-cluster plots are not always linear over the whole curve, and the exponent depends on the parameters. For large $\epsilon_{\mathrm{HH}} = 20k_BT$ the plots are roughly linear with fractal dimension $D_f \approx 2.5$, which is insensitive to the value of ϵ_{HC}. This is close to the value $D_f = 2$ observed in simulations of patchy particles (15). For smaller $\epsilon_{\mathrm{HH}} = 12k_BT$ there is not a single power law relationship between R_g and M, but for large clusters $D_f \approx 3$. The reason for this difference is likely due to the difference in the protein dynamics. For $\epsilon_{\mathrm{HH}} = 12k_BT$, proteins in clusters can dynamically rearrange to satisfy the maximum number of bonds, tending to adopt more space-filling shapes; at larger ϵ_{HH}, HP1-HP1 bonds persist for long times, leading to dynamically arrested fractal clusters.

A variation on the above scheme, where neighbours are determined by considering actual interactions between NTE and hinge beads, gives similar D_f values. Using the radius of the smallest cluster enclosing sphere instead of the radius of gyration gives slightly smaller D_f values, but with a similar difference between the two models.

11 LIMITED VALENCE MODEL: ALTERNATIVE QUENCHING AND ADDITIONAL FIGURES.

As noted above and in the main text, the limited valence model behaves similarly to patchy particles in that the system can adopt long long lived metastable states with multiple fractal clusters (including "closed loops" where all bonds are satisfied). The observed structures therefore depend on the initial condition or the quenching procedure used. To highlight this, in Fig. S12(a-b) we show configurations obtained with two different quenches. In Fig. S12(a), after stating from an equilibrium configuration for $\epsilon_{\mathrm{HH}}, \epsilon_{\mathrm{HC}} = 0$, first the HP1-chromatin attraction was switched on, then later the HP1-HP1 attraction was switched on. This procedure generated structures where most of the proteins were associated with the polymer, and were spread roughly uniformly along it; a few small (closed loop) clusters were not associated with the polymer. In Fig. S12(b), first the HP1-HP1 attraction was switched on, and then later HP1-chromatin attractions were switched on. In this case the proteins tend to sit in larger clumps associates with smaller sections of the polymer; therefore, much larger polymer regions are left without proteins bound. This latter morphology arises because the large HP1 clusters form first, only later becoming associated with the polymer. In all other limited valence simulations in this work we switched on both interactions at the same time. To demonstrate that the limited valence HP1 can form a gel, we also performed a simulation with periodic boundaries and a smaller box size (higher HP1 density); a snapshot is shown in Fig. S12(c).

In Figs. S13-S15 we present some additional measurements for the limited valence model HP1s. In Fig. S13 we show the limited valence model phase diagram, drawing the crossover (or transition) lines between different regimes (or phases) in the same way as the multivalent case. We set the separation between the mixed and the fractal/absorbing clusters phases (orange line) where $\phi_{\mathrm{sep}} \approx 0.5$. As in the multivalent model, within the mixed regime ϕ_{sep} is approximately independent of ϵ_{HC}; we therefore define the coating regime as where ϕ_{sep} starts to increase with ϵ_{HC} (dashed green line). Interestingly, within the mixed regime ϕ_{sep} shows more of a dependence on ϵ_{HH} for the limited valence model than it did for the multivalent HP1s. Again similar to the multivalent case, for larger ϵ_{HH} the absorbing clusters phase is defined as where the fraction of HP1s bound to chromatin $f_{\mathrm{tot}} \geq 0.5$ [see Fig. S14]. In Fig. S15 the fraction of chromatin beads bound to HP1s and the radius of gyration are shown. Note the non-monotonic behaviour of f_c and R_g is similar to the multivalent case.

12 PROTEIN DYNAMICS

As detailed in the main text, to quantify protein dynamics we consider the bond-bond correlation function $\nu_{\mathrm{HH}}(\Delta t)$, which measures the proportion of HP1s which retain the same interaction partners after a time interval Δt. Figures S16(a)i-iii show example plots of $\nu_{\mathrm{HH}}(\Delta t)$ for different values of ϵ_{HH} and ϵ_{HC} for the multivalent protein model. Clearly, these do not show a simple exponential decay (there is not a straight line on a log-linear plot); for small interaction energies ν_{HH} decays quickly to very small values, while for large energies there is a fast initial decay followed by a much

slower decrease. For cases where $\epsilon_{\rm HH}$ and $\epsilon_{\rm HC}$ are both large the $\nu_{\rm HH}$ curve almost plateaus at long time intervals [Fig. S16(a)iii]. Similar curves are shown in Figs. S16(b)i-iii for the limited valence HP1 model.

The non-exponential form of the $\nu_{\rm HH}(\Delta t)$ curves suggest that there are multiple time scales involved in this decorrelation. For example specific hinge-NTE bonds might break and form on short time scales, while repositioning of an HP1 with respect to its neighbours might take longer. We tried fitting several functional forms to the $\nu_{\rm HH}(\Delta t)$, finding a sum of three exponentials to consistently give good fits:

$$\nu_{\rm HH}(t) = \frac{{\rm e}^{-t/\tau_0} + a_1 {\rm e}^{-t/\tau_1} + a_2 {\rm e}^{\tau_2}}{1 + a_1 + a_2}, \quad (9)$$

with decay times τ_0, τ_1, τ_2, and dimensionless constants a_1 and a_2. We do not assign any specific meaning to these times or constants, but with analogy to a single exponential, we identify the integral of $t \times \nu_{\rm HH}(t)$ over t as the mean decorrelation time

$$\tau_{\rm HH} = \int_0^\infty t \, \frac{{\rm e}^{-t/\tau_0} + a_1 {\rm e}^{-t/\tau_1} + a_2 {\rm e}^{\tau_2}}{1 + a_1 + a_2} \, {\rm d}t = \frac{\tau_0 + a_1\tau_1 + a_2\tau_2}{1 + a_1 + a_2}.$$

It is also common to fit a decorrelation function to a stretched exponential ($\exp[-(t/\tau_0)^\beta]$ with $\beta < 1$), but we found this only gave a good fit for a few specific interaction energies. Grey lines in Figs. S16(a)i-iii and Figs. S16(b)i-iii show fits to the function in Eq. (9). The fits are good except for parameters corresponding to the mixed phase [lower curves in Figs. S16(a)i and (b)i], where $\nu_{\rm HH}(\Delta t)$ drops quickly to very small values and the error bars are large.

In Figs. S16(a)iv-v and Figs. S16(b)iv-v we show how $\tau_{\rm HH}$ varies with the interaction energies. This is similar to Figs. 6(b-c) in the main text, except here we use the effective (re-scaled) energies $E_{\rm HH}$ and $E_{\rm HC}$, as defined in Supporting text, section 4; we note there is very little difference in the shape of the curves. Figures S16(a)vi and (b)vi show the variation of $\tau_{\rm HH}$ with both energies as heat maps, with the boundaries between the regimes overlaid; this highlights some similarities and differences between the two models. In both cases, as expected $\tau_{\rm HH}$ is very small in the mixed phase. For $\epsilon_{\rm HC}$ and $\epsilon_{\rm HH}$ just large enough to enter the droplet/cluster phase, in both models $\tau_{\rm HH}$ is of the order $5 \times 10^2\tau$, and the droplet/clusters are highly dynamic on the time scale of the simulations (despite their different morphologies). Both models show a roughly order of magnitude increase in $\tau_{\rm HH}$ as $\epsilon_{\rm HC}$ is increased and chromatin becomes absorbed. The models differ substantially only at higher $\epsilon_{\rm HH}$ values: in the limited valence case there is no longer a large slow-down as the polymer becomes absorbed. It is interesting to note that although the limited valence model displays clusters with a fractal/gel-like morphology usually associated with arrested dynamics, the bond-bond decorrelation time is similar to the multivalent case. In this sense both models display similarly "arrested dynamics", but while all configurations of the multivalent proteins look similar macroscopically (a spherical droplet), the limited valence fractal clusters have a macroscopic appearance which depends on their history.

13 EFFECT OF POLYMER AND PROTEIN MODEL DETAILS

To check he robustness of our results, we perform several test simulations for similar systems, but changing some of the fine details of the chromatin and polymer models.

First, we consider a stiffer polymer, increasing the persistence length from $l_p = 4\,\sigma$ to $l_p = 8\,\sigma$ [this is still within the relevant range for chromatin, see e.g., (16)]. We have simulated systems with interaction parameter values on either side of the boundary between the coating and absorbed droplet regimes, and either side of the boundary between the mixed and coating regimes (Fig. S18). As expected, the general behaviour within each regimes is not affected by the increased stiffness. There is no difference, on average, in the proportions of HP1s binding in the bridging, coating and dangling modes [Figs. S18(a) and (d)]. There is a slight change in the fraction of polymer beads bound by HP1, f_c, at the coating/absorbing droplet crossover [Fig. S18(b)]; in the latter regime, polymer loops emerge from the droplet, and a higher energy cost for bending will favour longer loops. Consistent with this, the radius of gyration also increases slightly here [Fig. S18(c)]. At the mixed/coating crossover, f_c is not affected by the persistence length, since for a swollen polymer, this increase in stiffness does not modify the affinity of the HP1s. However, as expected for a stiffer polymer, there is a clear increase of the radius of gyration compared to the previous simulations (by about 10%).

Second, we consider a modified version the multivalent protein model were the HP1 dimer is flexible rather than a rigid body. Since the hinge domain is disordered and therefore flexible, one might expect the molecule to be able to bend at this point. The simplest way to represent this in the simulations is to have each dimer represented by three rigid bodies: one which contains just a CSD bead, and two which each contain one hinge, one NTE, and one CD bead. The hinges are then connected to the CSD using a harmonic bond described by

$$U_{\rm HARM}(r) = K(r - r_0)^2,$$

where r is the separation of the bonded hinge and CSD, K is the bond strength, and the equilibrium distance $r_0 = 0.5\,\sigma$. This allows free rotation about the hinge. All other protein-protein and protein-chromatin interactions were kept the same. We then performed simulations at several points in the phase diagram. Strikingly, we observed the same behaviour as in the previous rigid HP1 simulations. Importantly, we still found that for $\epsilon_{\rm HH} = 4\ k_BT$, when $\epsilon_{\rm HC}$ is small no droplet forms, but when $\epsilon_{\rm HC}$ is large, binding to the polymer and the bridging induced attraction leads to droplet formation. In all of the regimes studied, there were some small changes in values of quantities such at the fraction of HP1s binding in the different modes, and the fraction of polymer beads bound. This suggests that the positions of the boundaries between the regimes have shifted slightly, but the qualitative behaviour is unchanged. A crucial determinant of the coating behaviour is that the protein's shape allows binding in the coating mode without, e.g., a deformation to the chromatin which costs energy; the flexible HP1 dimer model in fact allows coating more readily, and we do see a slight increase in binding in this mode.

REFERENCES

1. Brackley, C. A., S. Taylor, A. Papantonis, P. R. Cook, and D. Marenduzzo, 2013. Nonspecific bridging-induced attraction drives clustering of DNA-binding proteins and genome organization. *Proc. Natl. Acad. Sci. USA* 110:E3605–E3611.

2. Larson, A. G., D. Elnatan, M. M. Keenen, M. J. Trnka, J. B. Johnston, A. L. Burlingame, D. A. Agard, S. Redding, and G. J. Narlikar, 2017. Liquid droplet formation by HP1α suggests a role for phase separation in heterochromatin. *Nature* 547:236–240.

3. Lomberk, G., L. Wallrath, and R. Urrutia, 2006. The Heterochromatin Protein 1 family. *Genome Biology* 7:228.

4. Plimpton, S., 1995. Fast Parallel Algorithms for Short-Range Molecular Dynamics. *J. Comp. Phys.* 117:1–19.

5. Mel'nikov, V. I., 1991. The Kramers problem: Fifty years of development. *Physics Reports* 209:1–71.

6. Brackley, C. A., 2020. Polymer compaction and bridging-induced clustering of protein-inspired patchy particles. *J. Phys. Condens. Matter* 32:314002.

7. Bryan, L. C., D. R. Weilandt, A. L. Bachmann, S. Kilic, C. C. Lechner, P. D. Odermatt, G. E. Fantner, S. Georgeon, O. Hantschel, V. Hatzimanikatis, and B. Fierz, 2017. Single-molecule kinetic analysis of HP1-chromatin binding reveals a dynamic network of histone modification and DNA interactions. *Nucleic Acids Research* 45:10504–10517.

8. Sindhikara, D., Y. Meng, and A. E. Roitberg, 2008. Exchange frequency in replica exchange molecular dynamics. *J. Chem. Phys.* 128:01B609.

9. Russo, J., P. Tartaglia, and F. Sciortino, 2009. Reversible gels of patchy particles: role of the valence. *J. Chem. Phys.* 131:014504.

10. Sciortino, F., and E. Zaccarelli, 2011. Reversible gels of patchy particles. *Current Opinion in Solid State and Materials Science* 15:246–253.

11. Bianchi, E., R. Blaak, and C. N. Likos, 2011. Patchy colloids: state of the art and perspectives. *Physical Chemistry Chemical Physics* 13:6397–6410.

12. Lindquist, B. A., R. B. Jadrich, D. J. Milliron, and T. M. Truskett, 2016. On the formation of equilibrium gels via a macroscopic bond limitation. *J. Chem. Phys.* 145:074906.

13. Jungblut, S., J.-O. Joswig, and A. Eychmüller, 2019. Diffusion- and reaction-limited cluster aggregation revisited. *Physical Chemistry Chemical Physics* 21:5723–5729.

14. Wu, H., M. Lattuada, and M. Morbidelli, 2013. Dependence of fractal dimension of DLCA clusters on size of primary particles. *Advances in Colloid and Interface Science* 195-196:41–49.

15. Audus, D. J., F. W. Starr, and J. F. Douglas, 2018. Valence, loop formation and universality in self-assembling patchy particles. *Soft Matter* 14.

16. Langowski, J., 2006. Polymer chain models of DNA and chromatin. *The European Physical Journal E* 19:241–249.
